# Supplementary material for: Modulation of Early Neutrophil Granulation: The Circulating Tumor Cell-Extravesicular Connection in Pancreatic Ductal Adenocarcinoma
Source: Cancers (Basel). 2021 May 31;13(11):2727. doi: 10.3390/cancers13112727 (PMC8198339; doi:10.3390/cancers13112727)
Supplement: Supplementary file 1 [file cancers-13-02727-s001.zip › cancers-1202292-supplementary/cancers-1202292-supplementary figures_053121.pdf]

cancers

Supplementary Materials: Modulation of Early Neutrophil Granulation: The Circulating Tumor Cell-Extravesicular Connection in Pancreatic Ductal Adenocarcinoma

Harrys Kishore Charles Jacob, John Lalith Charles Richard, Rossana Signorelli, Tyler Kashuv, Shweta Lavania, Utpreksha Vaish, Ranjitha Boopathy, Ashley Middleton, Melinda Minucci Boone, Ramakrishnan Sundaram, Vikas Dudeja and Ashok Kumar Saluja

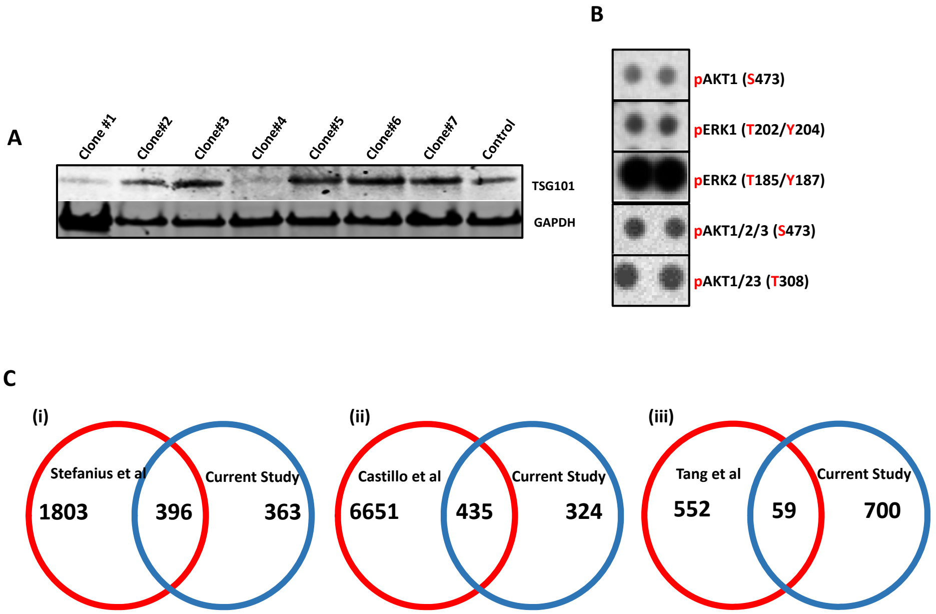

Figure S1: Western blots of CM61 cell lines assaying for knockdown and phosphorylation status. **A.** Clones assayed for silencing of *TSG101*; **B**. phosphoMAPK arrays indicating conservation of phosphorylation and **C**. comparison of other published studies depicting the uniqueness of the CTC EVome.

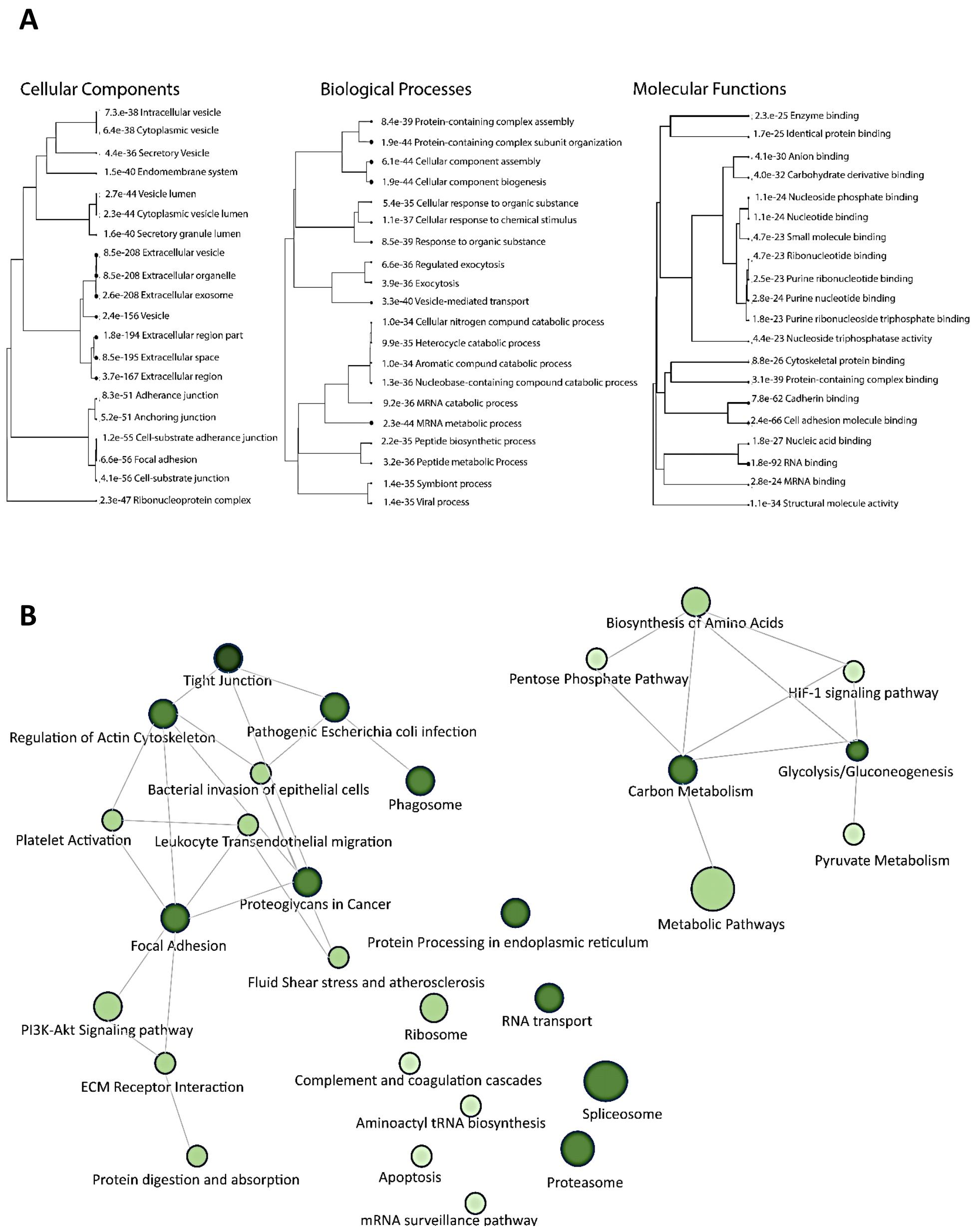

Figure S2: Gene Ontology and pathway enrichment analysis of proteins enriched from the CTC lines. **A**. GO classification of the EVome of the CTCs and **B**. Common pathways that are enriched.

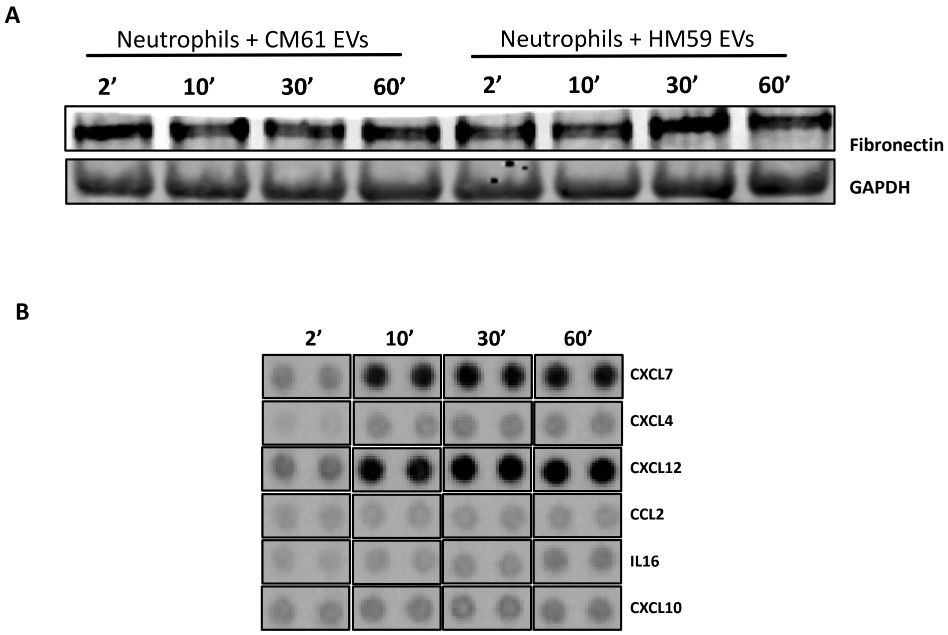

Figure S3: Western blots to assay secreted fibronectin and chemokines from neutrophils treated with CM61 CTC EVs. A. Secreted fibronectin from neutrophils treated with EVs. B. Chemokine levels in the neutrophil secretome.

Tables S1–S5 and Files S1–S2, you can check in the document file.

Conflicts of Interest: A.K.S. is one of the inventors of Minnelide, which has been licensed to Minneamrita Therapeutics by the University of Minnesota; and is its cofounder and CSO. The rest of the authors declare no conflict of interest.
