# Supplementary material for: Modulation of Early Neutrophil Granulation: The Circulating Tumor Cell-Extravesicular Connection in Pancreatic Ductal Adenocarcinoma
Source: Cancers (Basel). 2021 May 31;13(11):2727. doi: 10.3390/cancers13112727 (PMC8198339; doi:10.3390/cancers13112727)
Supplement: Supplementary file 1 [file cancers-13-02727-s001.zip › cancers-1202292-supplementary/Supplementary File S1.pdf]

NANOSIGHT

CM61 1 in 10 2021-05-11 10-55-08

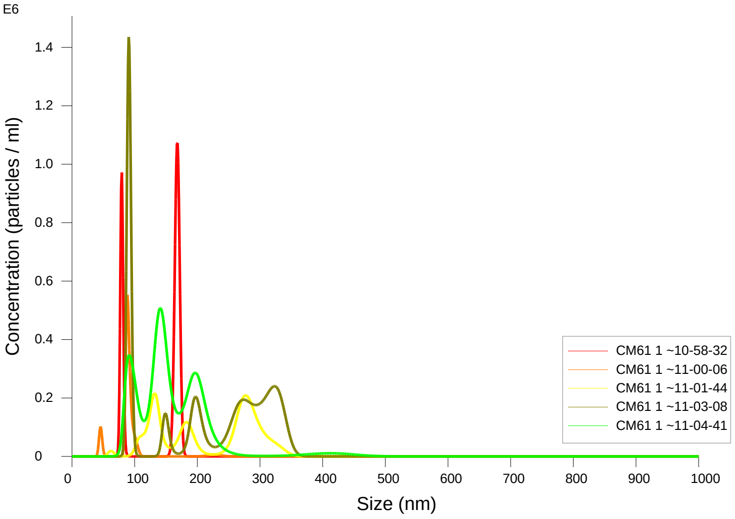

FTLA Concentration / Size graph for Experiment:
CM61 1 in 10 2021-05-11 10-55-08

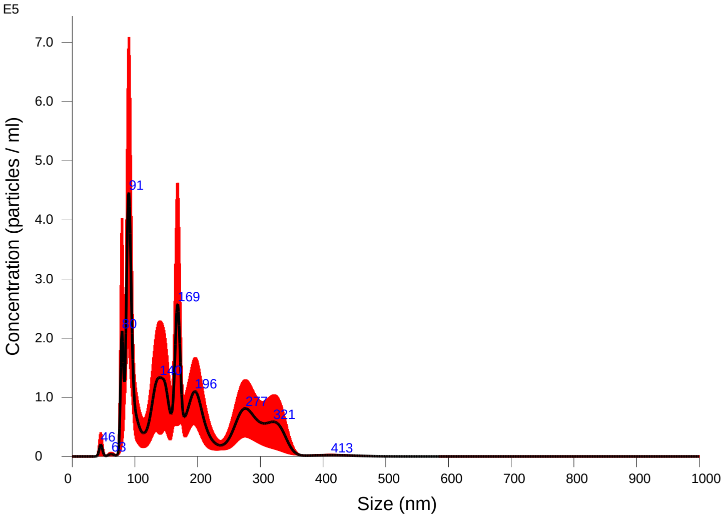

Averaged FTLA Concentration / Size for Experiment:
CM61 1 in 10 2021-05-11 10-55-08
Error bars indicate + / -1 standard error of the mean

Included Files

CM61 1 in 10 2021-05-11 10-58-32
CM61 1 in 10 2021-05-11 11-00-06
CM61 1 in 10 2021-05-11 11-01-44
CM61 1 in 10 2021-05-11 11-03-08
CM61 1 in 10 2021-05-11 11-04-41

Details

NTA Version:	NTA 3.4 Build 3.4.003
Script Used:	SOP Standard Measurement 10-55-08AM 11May2021.txt
Time Captured:	10:55:08 11/05/2021
Operator:	
Pre-treatment:	
Sample Name:	CM61 1 in 10
Diluent:	water
Remarks:	

Capture Settings

Camera Type:	sCMOS
Laser Type:	Blue488
Camera Level:	10 (NTA 3.0 Levels)
Slider Shutter:	600
Slider Gain:	250
FPS	25.0
Number of Frames:	1498
Temperature:	24.7 - 24.9 ºC
Viscosity:	(Water) 0.891 - 0.894 cP
Dilution factor:	Dilution not recorded

Analysis Settings

Detect Threshold:	5
Blur Size:	Auto
Max Jump Distance:	Auto: 12.9 - 22.9 pix

Results

Stats: Merged Data

Mean:	179.5 nm
Mode:	90.1 nm
SD:	81.7 nm
D10:	87.7 nm
D50:	166.0 nm
D90:	304.7 nm

Stats: Mean +/- Standard Error

Mean:	163.1 +/- 23.9 nm
Mode:	124.0 +/- 15.3 nm
SD:	60.0 +/- 11.8 nm
D10:	86.5 +/- 11.8 nm
D50:	174.0 +/- 27.7 nm
D90:	223.2 +/- 42.1 nm
Concentration:	2.39e+07 +/- 6.44e+06 particles/ml
	1.3 +/- 0.4 particles/frame
	1.7 +/- 0.6 centres/frame

CM61 1 in 10 2021-05-11 10-55-08

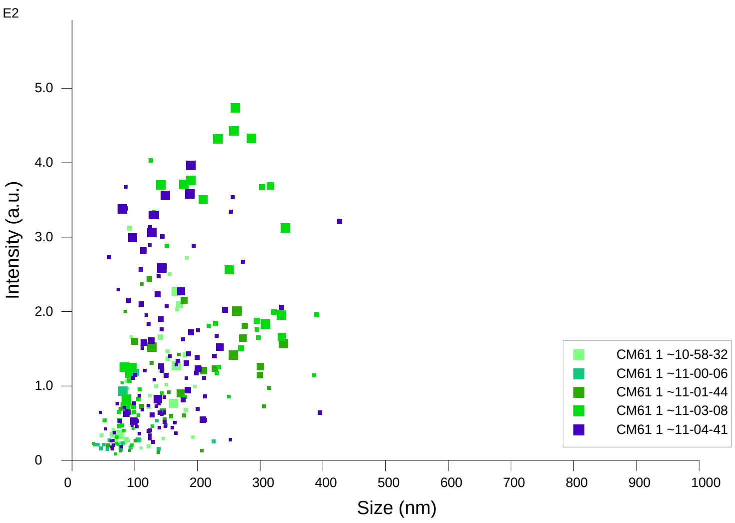

Intensity / Size graph for Experiment:
CM61 1 in 10 2021-05-11 10-55-08

NANOSIGHT

CF49ConcUndil 2021-05-11 11-51-33

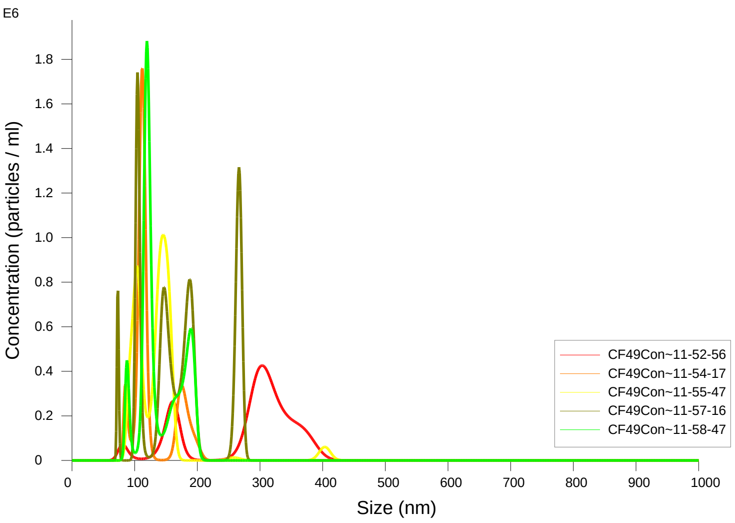

FTLA Concentration / Size graph for Experiment:
CF49ConcUndil 2021-05-11 11-51-33

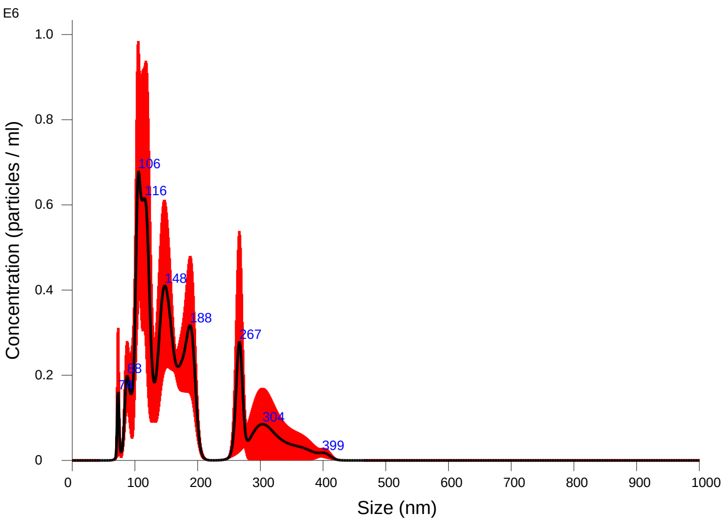

Averaged FTLA Concentration / Size for Experiment:
CF49ConcUndil 2021-05-11 11-51-33
Error bars indicate + / -1 standard error of the mean

Included Files

CF49ConcUndil 2021-05-11 11-52-56
CF49ConcUndil 2021-05-11 11-54-17
CF49ConcUndil 2021-05-11 11-55-47
CF49ConcUndil 2021-05-11 11-57-16
CF49ConcUndil 2021-05-11 11-58-47

Details

NTA Version:	NTA 3.4 Build 3.4.003
Script Used:	SOP Standard Measurement 11-51-33AM 11May2021.txt
Time Captured:	11:51:33 11/05/2021
Operator:	
Pre-treatment:	
Sample Name:	CF49 fresh undil
Diluent:	water
Remarks:	

Capture Settings

Camera Type:	sCMOS
Laser Type:	Blue488
Camera Level:	10 (NTA 3.0 Levels)
Slider Shutter:	600
Slider Gain:	250
FPS	25.0
Number of Frames:	1498
Temperature:	26.2 - 26.3 ºC
Viscosity:	(Water) 0.863 - 0.865 cP
Dilution factor:	Dilution not recorded

Analysis Settings

Detect Threshold:	5
Blur Size:	Auto
Max Jump Distance:	Auto: 13.8 - 20.3 pix

Results

Stats: Merged Data

Mean:	170.1 nm
Mode:	105.8 nm
SD:	75.5 nm
D10:	102.2 nm
D50:	147.8 nm
D90:	296.7 nm

Stats: Mean +/- Standard Error

Mean:	171.4 +/- 28.0 nm
Mode:	157.1 +/- 37.2 nm
SD:	51.7 +/- 9.2 nm
D10:	112.7 +/- 10.3 nm
D50:	169.1 +/- 34.7 nm
D90:	231.9 +/- 37.6 nm
Concentration:	4.88e+07 +/- 5.60e+06 particles/ml
	2.7 +/- 0.3 particles/frame
	3.4 +/- 0.3 centres/frame

CF49ConcUndil 2021-05-11 11-51-33

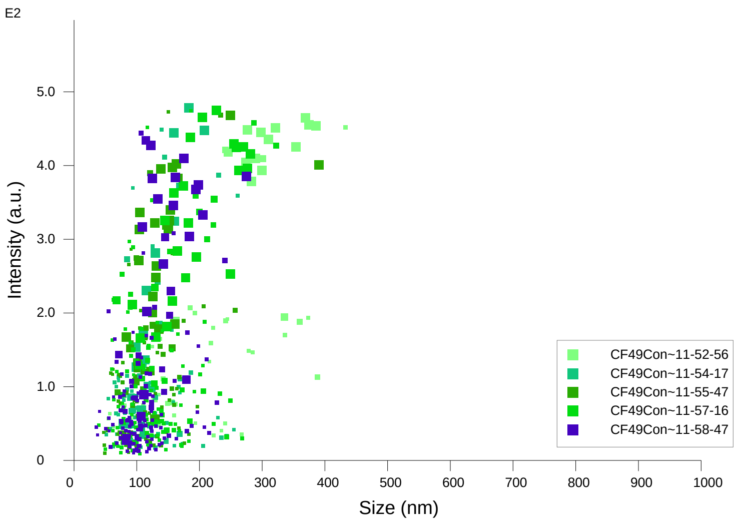

Intensity / Size graph for Experiment:
CF49ConcUndil 2021-05-11 11-51-33

NANOSIGHT

CF49SS 2021-05-11 12-21-15

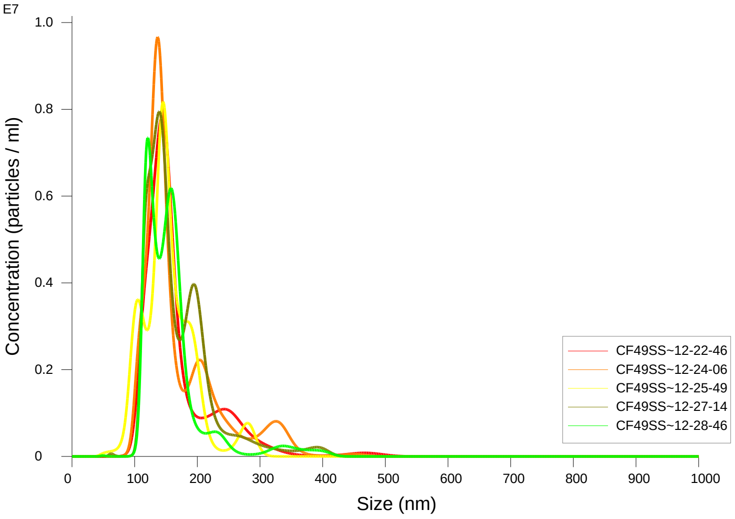

FTLA Concentration / Size graph for Experiment:
CF49SS 2021-05-11 12-21-15

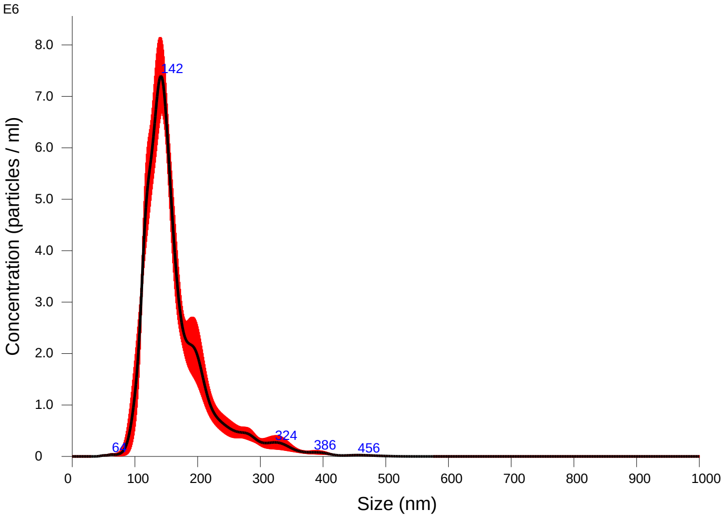

Averaged FTLA Concentration / Size for Experiment:
CF49SS 2021-05-11 12-21-15
Error bars indicate + / -1 standard error of the mean

Included Files

CF49SS 2021-05-11 12-22-46
CF49SS 2021-05-11 12-24-06
CF49SS 2021-05-11 12-25-49
CF49SS 2021-05-11 12-27-14
CF49SS 2021-05-11 12-28-46

Details

NTA Version:	NTA 3.4 Build 3.4.003
Script Used:	SOP Standard Measurement 12-21-15PM 11May2021.txt
Time Captured:	12:21:15 11/05/2021
Operator:	
Pre-treatment:	
Sample Name:	CF49SS fresh undil
Diluent:	water
Remarks:	

Capture Settings

Camera Type:	sCMOS
Laser Type:	Blue488
Camera Level:	10 (NTA 3.0 Levels)
Slider Shutter:	600
Slider Gain:	250
FPS	25.0
Number of Frames:	1498
Temperature:	26.6 - 26.7 ºC
Viscosity:	(Water) 0.855 - 0.858 cP
Dilution factor:	Dilution not recorded

Analysis Settings

Detect Threshold:	5
Blur Size:	Auto
Max Jump Distance:	Auto: 12.8 - 13.5 pix

Results

Stats: Merged Data

Mean:	164.3 nm
Mode:	141.5 nm
SD:	55.1 nm
D10:	115.7 nm
D50:	148.8 nm
D90:	232.6 nm

Stats: Mean +/- Standard Error

Mean:	164.0 +/- 3.2 nm
Mode:	137.3 +/- 4.4 nm
SD:	54.0 +/- 3.4 nm
D10:	114.9 +/- 2.8 nm
D50:	148.9 +/- 0.8 nm
D90:	230.0 +/- 12.2 nm
Concentration:	5.26e+08 +/- 2.27e+07 particles/ml
	28.7 +/- 1.2 particles/frame
	33.3 +/- 1.7 centres/frame

NANOSIGHT

CF49SS 2021-05-11 12-21-15

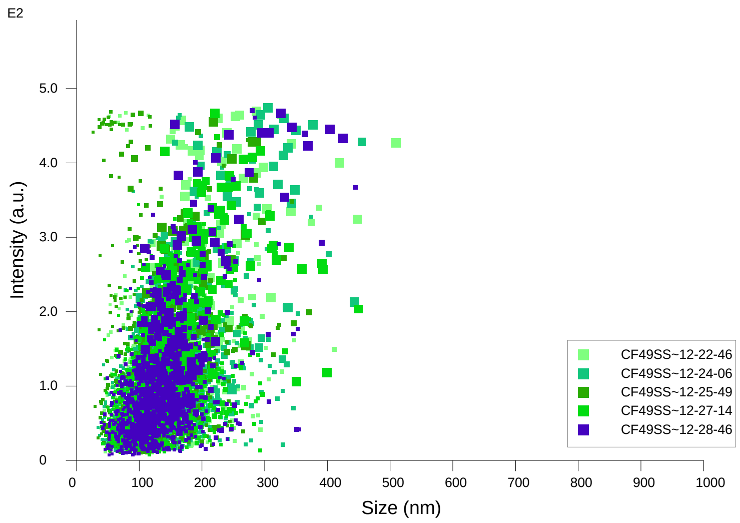

Intensity / Size graph for Experiment:
CF49SS 2021-05-11 12-21-15

NANOSIGHT

CF49SS+E 2021-05-11 12-37-38

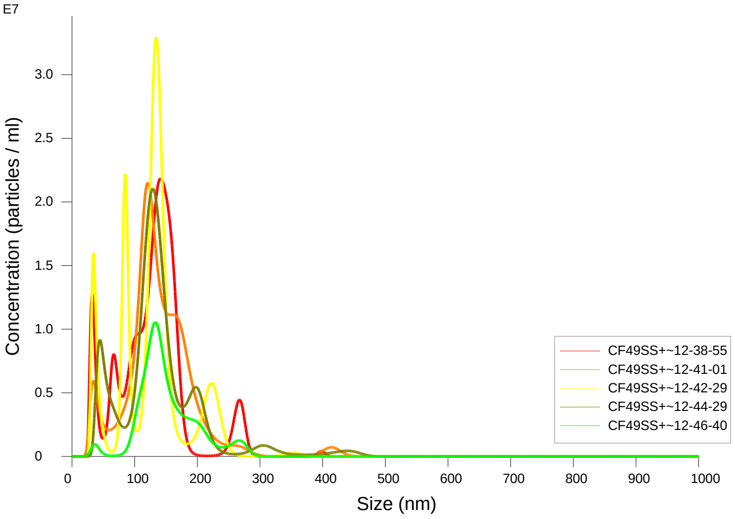

FTLA Concentration / Size graph for Experiment:
CF49SS+E 2021-05-11 12-37-38

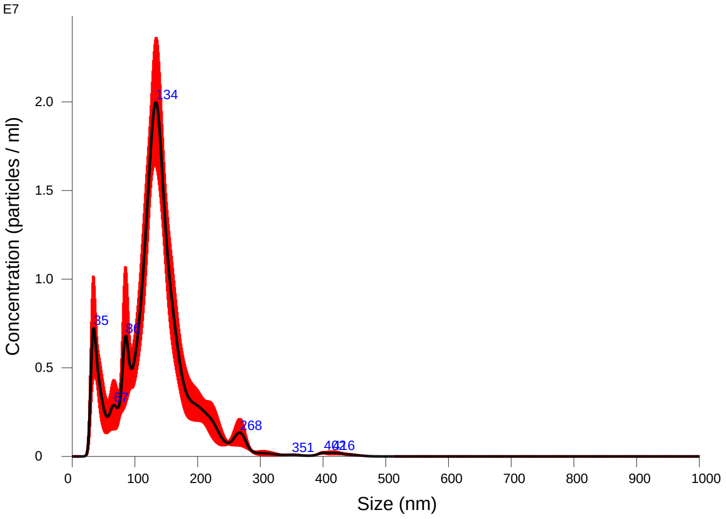

Averaged FTLA Concentration / Size for Experiment:
CF49SS+E 2021-05-11 12-37-38
Error bars indicate + / -1 standard error of the mean

Included Files

CF49SS+E 2021-05-11 12-38-55
CF49SS+E 2021-05-11 12-41-01
CF49SS+E 2021-05-11 12-42-29
CF49SS+E 2021-05-11 12-44-29
CF49SS+E 2021-05-11 12-46-40

Details

NTA Version:	NTA 3.4 Build 3.4.003
Script Used:	SOP Standard Measurement 12-37-38PM 11May2021.txt
Time Captured:	12:37:38 11/05/2021
Operator:	
Pre-treatment:	
Sample Name:	CF49SS+E fresh undil
Diluent:	water
Remarks:	

Capture Settings

Camera Type:	sCMOS
Laser Type:	Blue488
Camera Level:	10 (NTA 3.0 Levels)
Slider Shutter:	600
Slider Gain:	250
FPS	25.0
Number of Frames:	1498
Temperature:	26.8 - 26.8 ºC
Viscosity:	(Water) 0.853 - 0.854 cP
Dilution factor:	Dilution not recorded

Analysis Settings

Detect Threshold:	5
Blur Size:	Auto
Max Jump Distance:	Auto: 15.3 - 21.9 pix

Results

Stats: Merged Data

Mean:	135.3 nm
Mode:	133.4 nm
SD:	58.0 nm
D10:	62.4 nm
D50:	132.8 nm
D90:	201.4 nm

Stats: Mean +/- Standard Error

Mean:	137.3 +/- 4.4 nm
Mode:	131.7 +/- 3.4 nm
SD:	56.1 +/- 3.4 nm
D10:	68.2 +/- 11.2 nm
D50:	133.8 +/- 2.0 nm
D90:	199.7 +/- 8.0 nm
Concentration:	1.45e+09 +/- 1.86e+08 particles/ml
	79.1 +/- 10.2 particles/frame
	136.5 +/- 23.7 centres/frame

CF49SS+E 2021-05-11 12-37-38

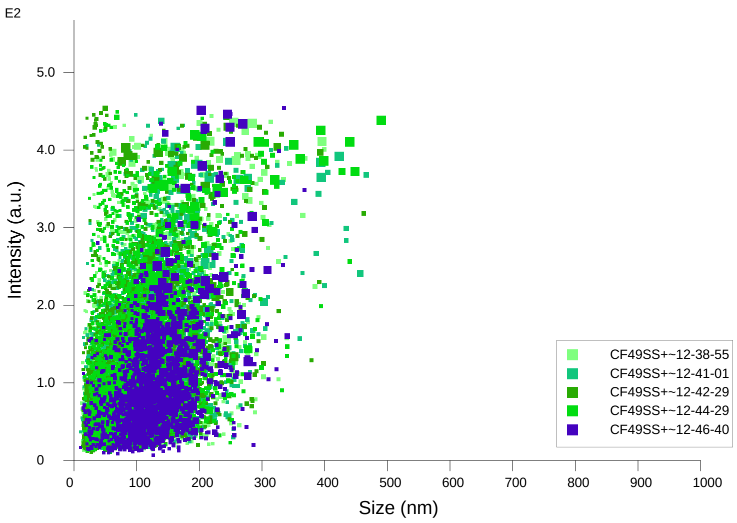

Intensity / Size graph for Experiment:
CF49SS+E 2021-05-11 12-37-38

NANOSIGHT

Hm59 Fresh Undil 2021-05-11 12-06-26

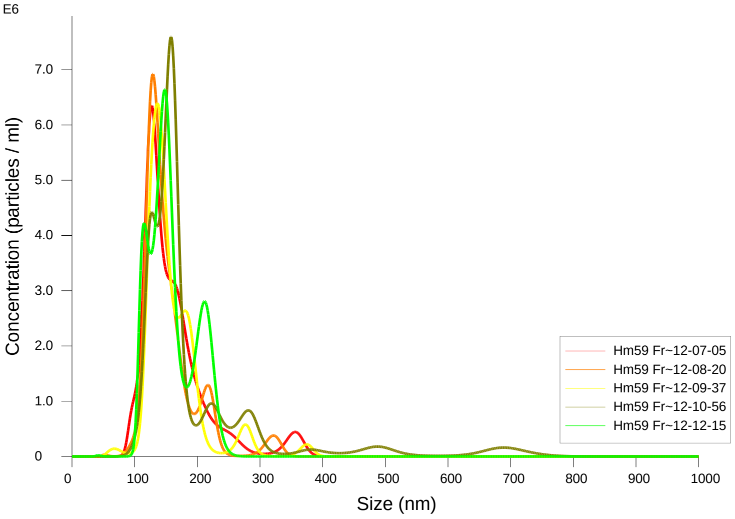

FTLA Concentration / Size graph for Experiment:
Hm59 Fresh Undil 2021-05-11 12-06-26

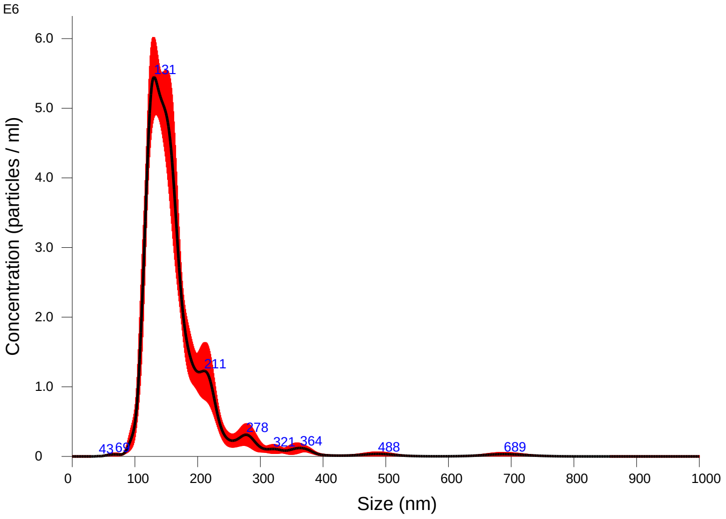

Averaged FTLA Concentration / Size for Experiment:
Hm59 Fresh Undil 2021-05-11 12-06-26
Error bars indicate + / -1 standard error of the mean

Included Files

Hm59 Fresh Undil 2021-05-11 12-07-05
Hm59 Fresh Undil 2021-05-11 12-08-20
Hm59 Fresh Undil 2021-05-11 12-09-37
Hm59 Fresh Undil 2021-05-11 12-10-56
Hm59 Fresh Undil 2021-05-11 12-12-15

Details

NTA Version:	NTA 3.4 Build 3.4.003
Script Used:	SOP Standard Measurement 12-06-26PM 11May2021.txt
Time Captured:	12:06:26 11/05/2021
Operator:	
Pre-treatment:	
Sample Name:	HM59 fresh undil
Diluent:	water
Remarks:	

Capture Settings

Camera Type:	sCMOS
Laser Type:	Blue488
Camera Level:	10 (NTA 3.0 Levels)
Slider Shutter:	600
Slider Gain:	250
FPS	25.0
Number of Frames:	1498
Temperature:	26.4 - 26.5 °C
Viscosity:	(Water) 0.859 - 0.861 cP
Dilution factor:	Dilution not recorded

Analysis Settings

Detect Threshold:	5
Blur Size:	Auto
Max Jump Distance:	Auto: 12.4 - 13.5 pix

Results

Stats: Merged Data

Mean:	167.5 nm
Mode:	130.6 nm
SD:	70.5 nm
D10:	118.7 nm
D50:	150.0 nm
D90:	224.2 nm

Stats: Mean +/- Standard Error

Mean:	166.4 +/- 7.6 nm
Mode:	139.7 +/- 5.9 nm
SD:	59.5 +/- 14.5 nm
D10:	118.9 +/- 1.7 nm
D50:	148.8 +/- 2.8 nm
D90:	229.1 +/- 16.0 nm
Concentration:	3.99e+08 +/- 1.85e+07 particles/ml
	21.8 +/- 1.0 particles/frame
	24.4 +/- 0.9 centres/frame

Hm59 Fresh Undil 2021-05-11 12-06-26

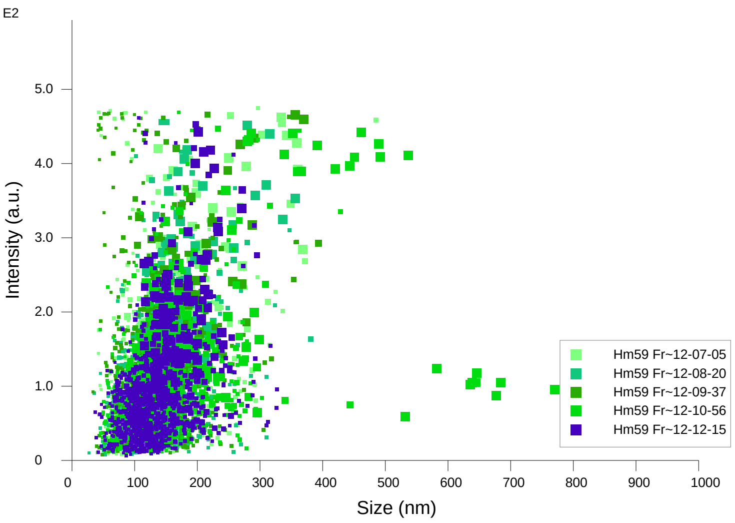

Intensity / Size graph for Experiment:
Hm59 Fresh Undil 2021-05-11 12-06-26

NANOSIGHT

Hm59 SS 2021-05-11 12-56-22

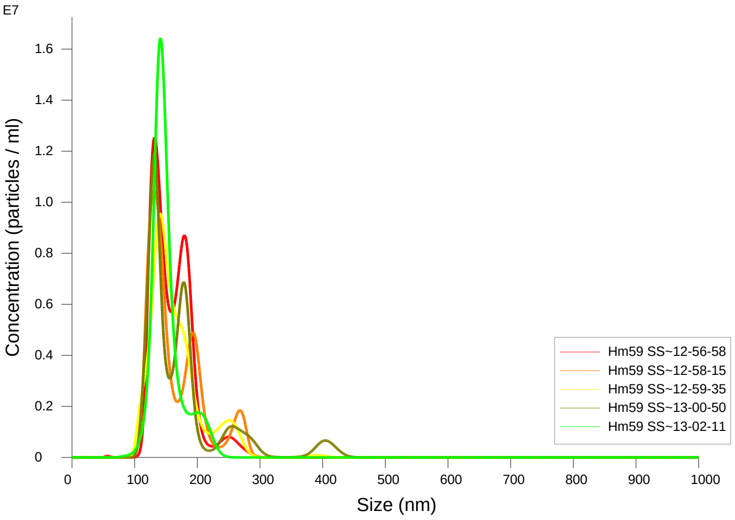

FTLA Concentration / Size graph for Experiment:
Hm59 SS 2021-05-11 12-56-22

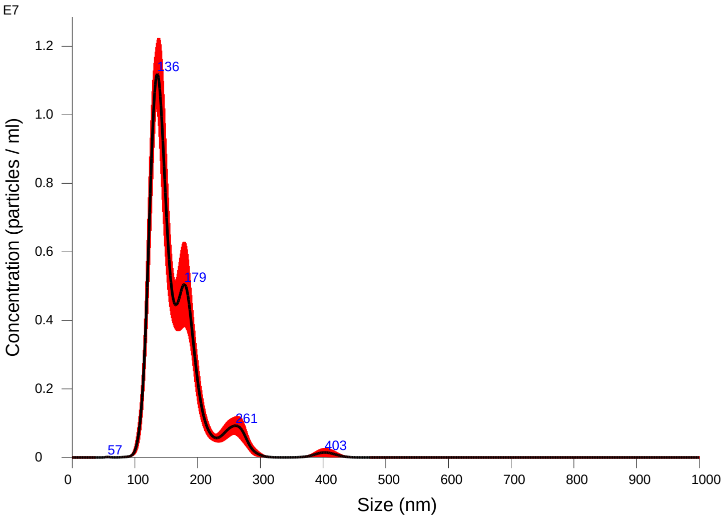

Averaged FTLA Concentration / Size for Experiment:
Hm59 SS 2021-05-11 12-56-22
Error bars indicate + / -1 standard error of the mean

Included Files

Hm59 SS 2021-05-11 12-56-58
Hm59 SS 2021-05-11 12-58-15
Hm59 SS 2021-05-11 12-59-35
Hm59 SS 2021-05-11 13-00-50
Hm59 SS 2021-05-11 13-02-11

Details

NTA Version:	NTA 3.4 Build 3.4.003
Script Used:	SOP Standard Measurement 12-56-22PM 11May2021.txt
Time Captured:	12:56:22 11/05/2021
Operator:	
Pre-treatment:	
Sample Name:	HM59 SS fresh undil
Diluent:	water
Remarks:	

Capture Settings

Camera Type:	sCMOS
Laser Type:	Blue488
Camera Level:	10 (NTA 3.0 Levels)
Slider Shutter:	600
Slider Gain:	250
FPS	25.0
Number of Frames:	1498
Temperature:	26.9 - 26.9 ºC
Viscosity:	(Water) 0.851 - 0.852 cP
Dilution factor:	Dilution not recorded

Analysis Settings

Detect Threshold:	5
Blur Size:	Auto
Max Jump Distance:	Auto: 12.1 - 13.0 pix

Results

Stats: Merged Data

Mean:	162.6 nm
Mode:	135.7 nm
SD:	44.2 nm
D10:	123.9 nm
D50:	149.5 nm
D90:	211.3 nm

Stats: Mean +/- Standard Error

Mean:	162.5 +/- 3.6 nm
Mode:	135.2 +/- 2.3 nm
SD:	41.6 +/- 6.8 nm
D10:	124.0 +/- 1.1 nm
D50:	150.7 +/- 1.9 nm
D90:	220.0 +/- 12.8 nm
Concentration:	6.25e+08 +/- 2.02e+07 particles/ml
	34.1 +/- 1.1 particles/frame
	37.8 +/- 1.0 centres/frame

NANOSIGHT

Hm59 SS 2021-05-11 12-56-22

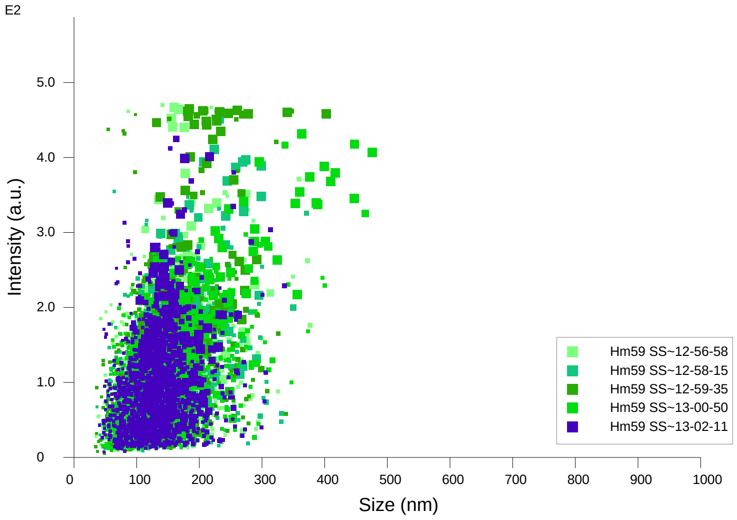

Intensity / Size graph for Experiment:
Hm59 SS 2021-05-11 12-56-22

NANOSIGHT

59SS+E 2021-05-11 13-09-29

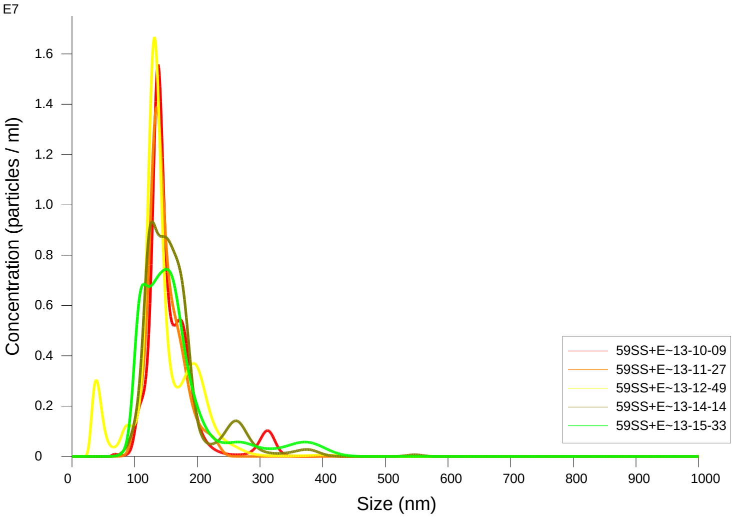

FTLA Concentration / Size graph for Experiment:
59SS+E 2021-05-11 13-09-29

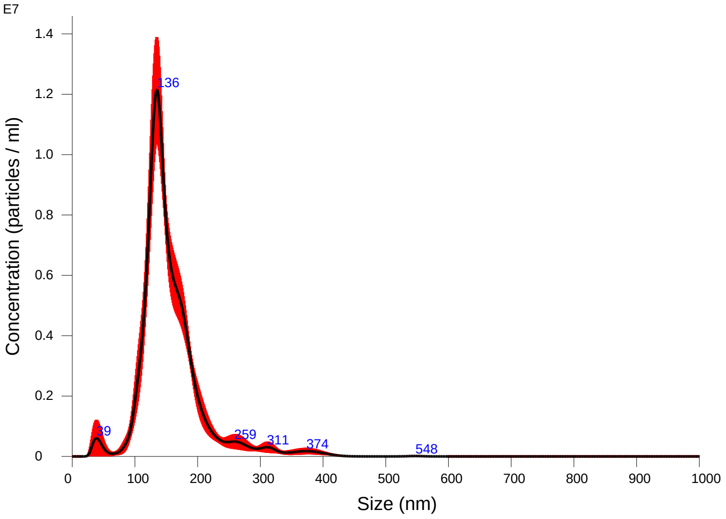

Averaged FTLA Concentration / Size for Experiment:
59SS+E 2021-05-11 13-09-29
Error bars indicate + / -1 standard error of the mean

Included Files

59SS+E 2021-05-11 13-10-09
59SS+E 2021-05-11 13-11-27
59SS+E 2021-05-11 13-12-49
59SS+E 2021-05-11 13-14-14
59SS+E 2021-05-11 13-15-33

Details

NTA Version:	NTA 3.4 Build 3.4.003
Script Used:	SOP Standard Measurement 01-09-29PM 11May2021.txt
Time Captured:	13:09:29 11/05/2021
Operator:	
Pre-treatment:	
Sample Name:	HM59 SS+E fresh undil
Diluent:	water
Remarks:	

Capture Settings

Camera Type:	sCMOS
Laser Type:	Blue488
Camera Level:	10 (NTA 3.0 Levels)
Slider Shutter:	600
Slider Gain:	250
FPS	25.0
Number of Frames:	1498
Temperature:	26.9 ºC
Viscosity:	(Water) 0.850 - 0.851 cP
Dilution factor:	Dilution not recorded

Analysis Settings

Detect Threshold:	5
Blur Size:	Auto
Max Jump Distance:	Auto: 12.8 - 17.3 pix

Results

Stats: Merged Data

Mean:	156.4 nm
Mode:	135.0 nm
SD:	51.5 nm
D10:	114.0 nm
D50:	144.8 nm
D90:	207.0 nm

Stats: Mean +/- Standard Error

Mean:	156.5 +/- 4.6 nm
Mode:	136.6 +/- 3.9 nm
SD:	48.4 +/- 6.5 nm
D10:	111.9 +/- 5.9 nm
D50:	145.6 +/- 2.9 nm
D90:	216.9 +/- 13.5 nm
Concentration:	7.30e+08 +/- 3.05e+07 particles/ml
	39.9 +/- 1.7 particles/frame
	57.9 +/- 14.5 centres/frame

59SS+E 2021-05-11 13-09-29

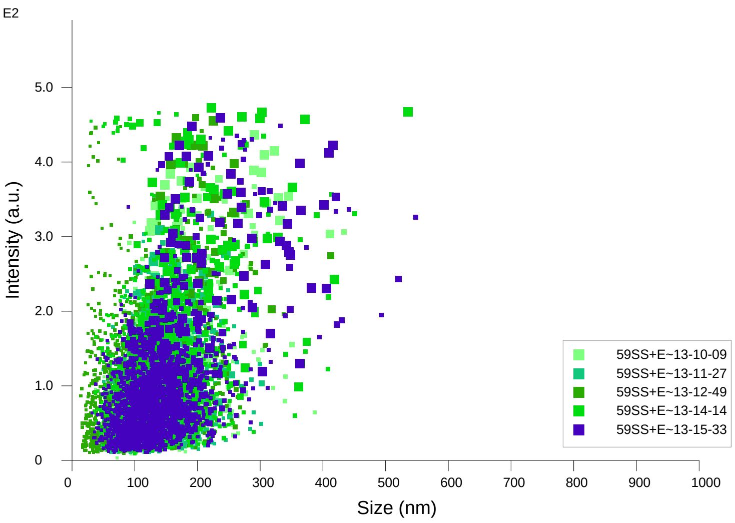

Intensity / Size graph for Experiment:
59SS+E 2021-05-11 13-09-29
