# Supplementary material for: Modulation of Early Neutrophil Granulation: The Circulating Tumor Cell-Extravesicular Connection in Pancreatic Ductal Adenocarcinoma
Source: Cancers (Basel). 2021 May 31;13(11):2727. doi: 10.3390/cancers13112727 (PMC8198339; doi:10.3390/cancers13112727)
Supplement: Supplementary file 1 [file cancers-13-02727-s001.zip › cancers-1202292-supplementary/Supplementary File S1.pptx]

## Slide 1
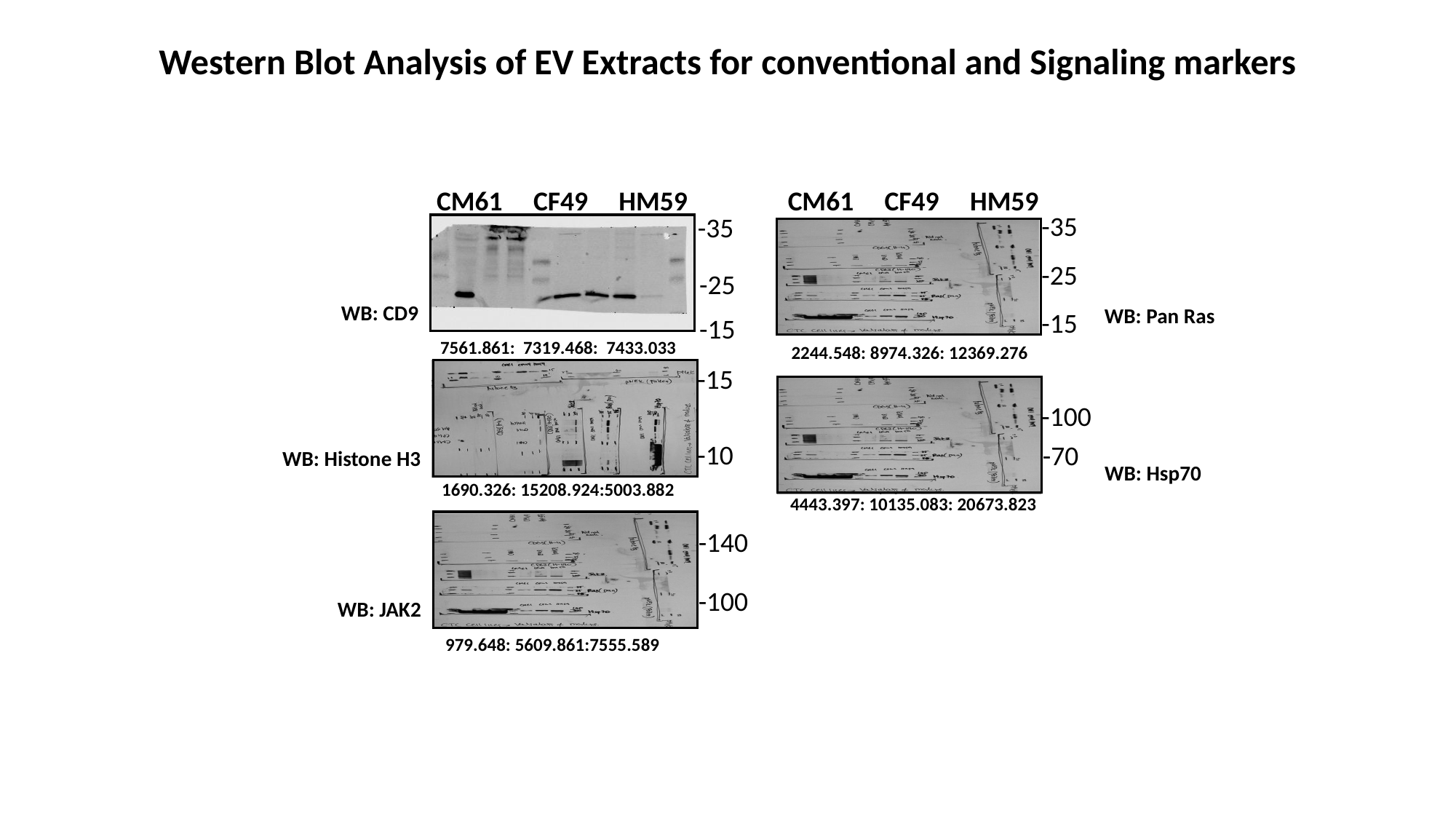

Western Blot Analysis of EV Extracts for conventional and Signaling markers
CM61 CF49 HM59
-35
-25
WB: CD9
-15
7561.861: 7319.468: 7433.033
-15
-10
WB: Histone H3
1690.326: 15208.924:5003.882
WB: JAK2
979.648: 5609.861:7555.589
CM61 CF49 HM59
-35
-25
-15
2244.548: 8974.326: 12369.276
-100
-70
WB: Hsp70
4443.397: 10135.083: 20673.823
WB: Pan Ras
-140
-100

## Slide 2
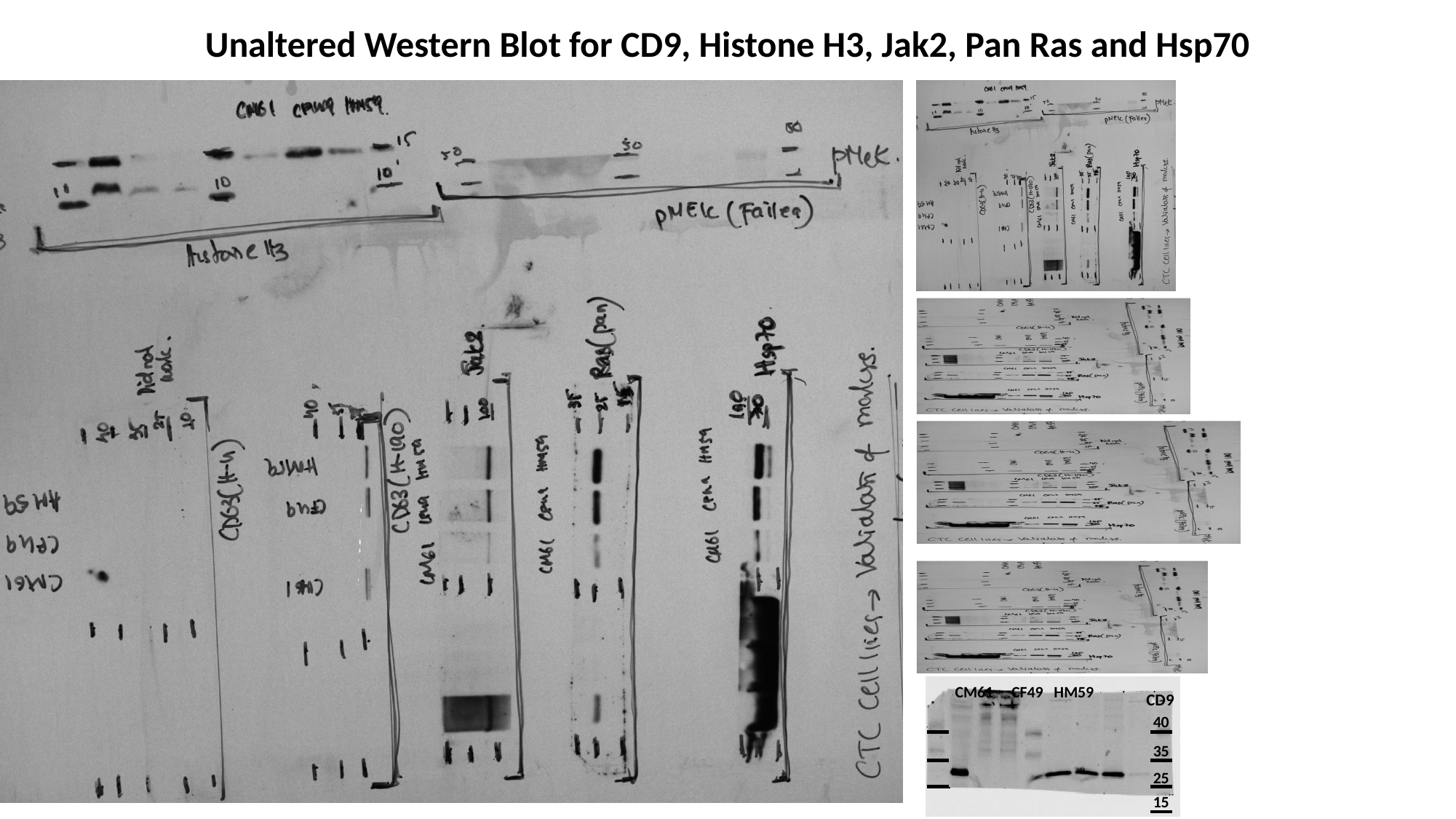

Unaltered Western Blot for CD9, Histone H3, Jak2, Pan Ras and Hsp70
 CM61 CF49 HM59
CD9
40
35
25
15

## Slide 3
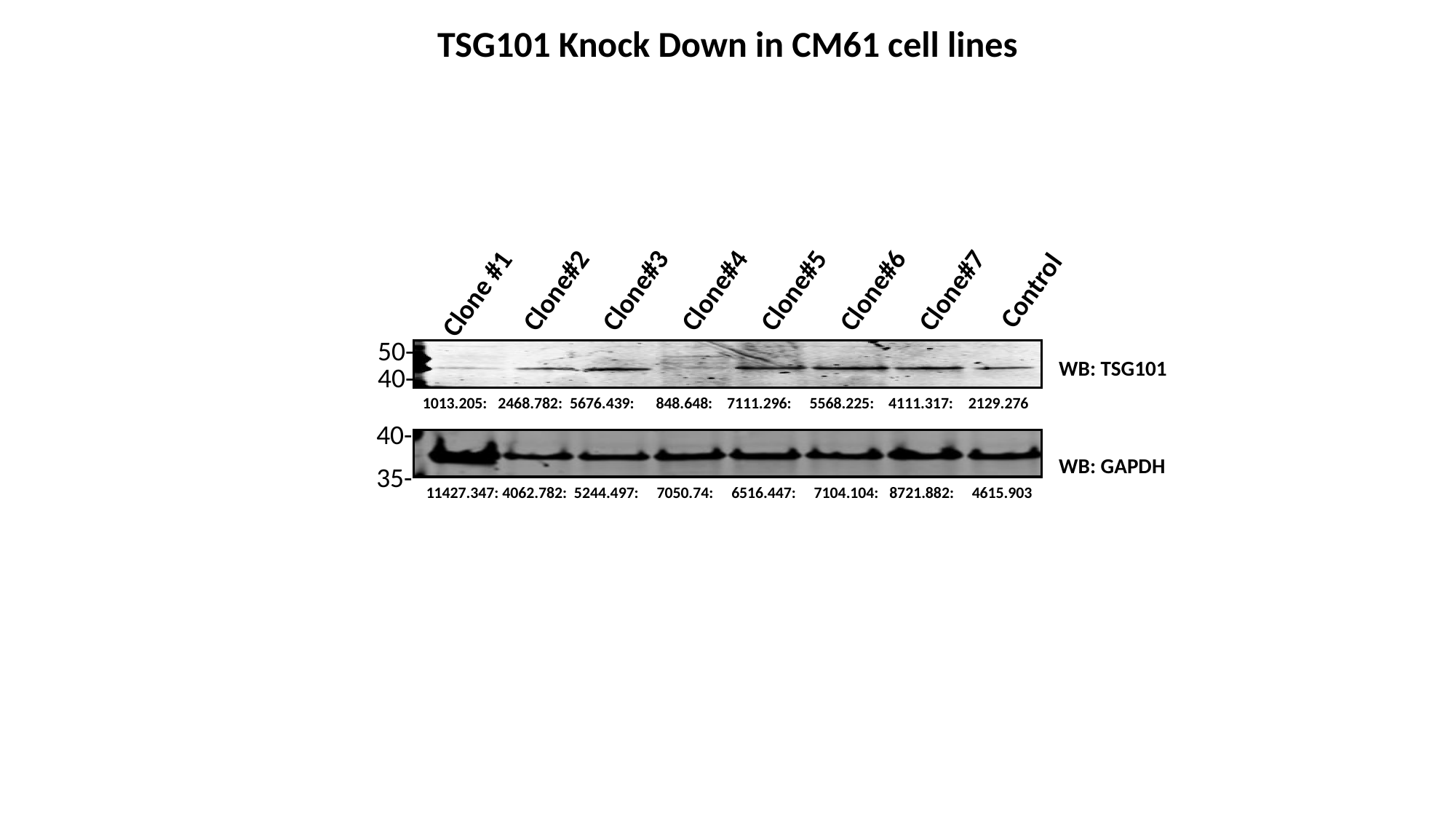

TSG101 Knock Down in CM61 cell lines
Clone#2
Clone#3
Clone#4
Clone#5
Clone#6
Clone#7
Control
Clone #1
50-
40-
WB: TSG101
1013.205: 2468.782: 5676.439: 848.648: 7111.296: 5568.225: 4111.317: 	2129.276
40-
35-
WB: GAPDH
11427.347: 4062.782: 5244.497: 7050.74: 6516.447: 7104.104: 8721.882:	4615.903

## Slide 4
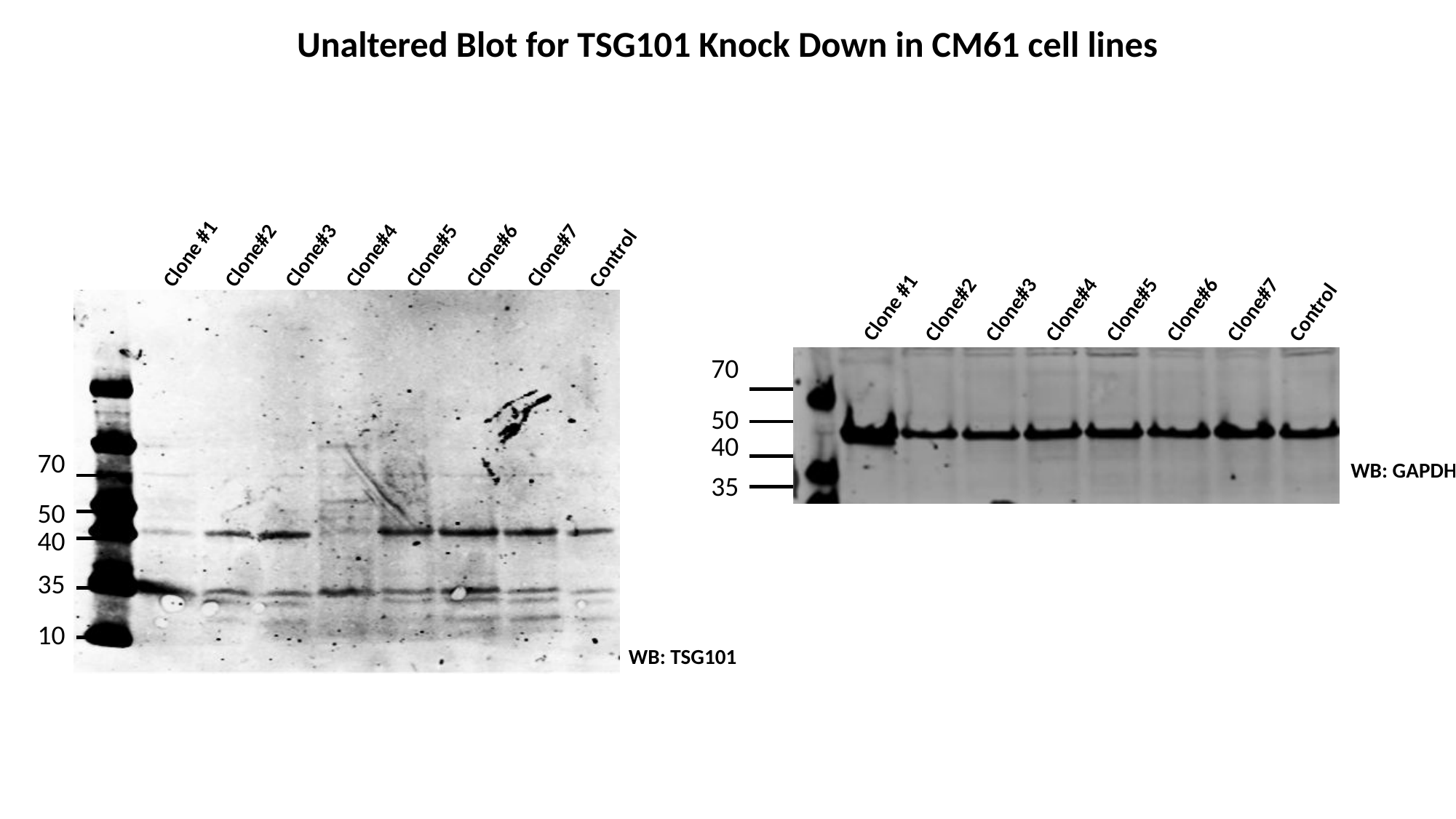

Unaltered Blot for TSG101 Knock Down in CM61 cell lines
Clone #1
Clone#2
Clone#3
Clone#4
Clone#5
Clone#6
Clone#7
Control
Clone #1
Clone#2
Clone#3
Clone#4
Clone#5
Clone#6
Clone#7
Control
70
50
40
35
WB: GAPDH
70
50
40
35
10
WB: TSG101

## Slide 5
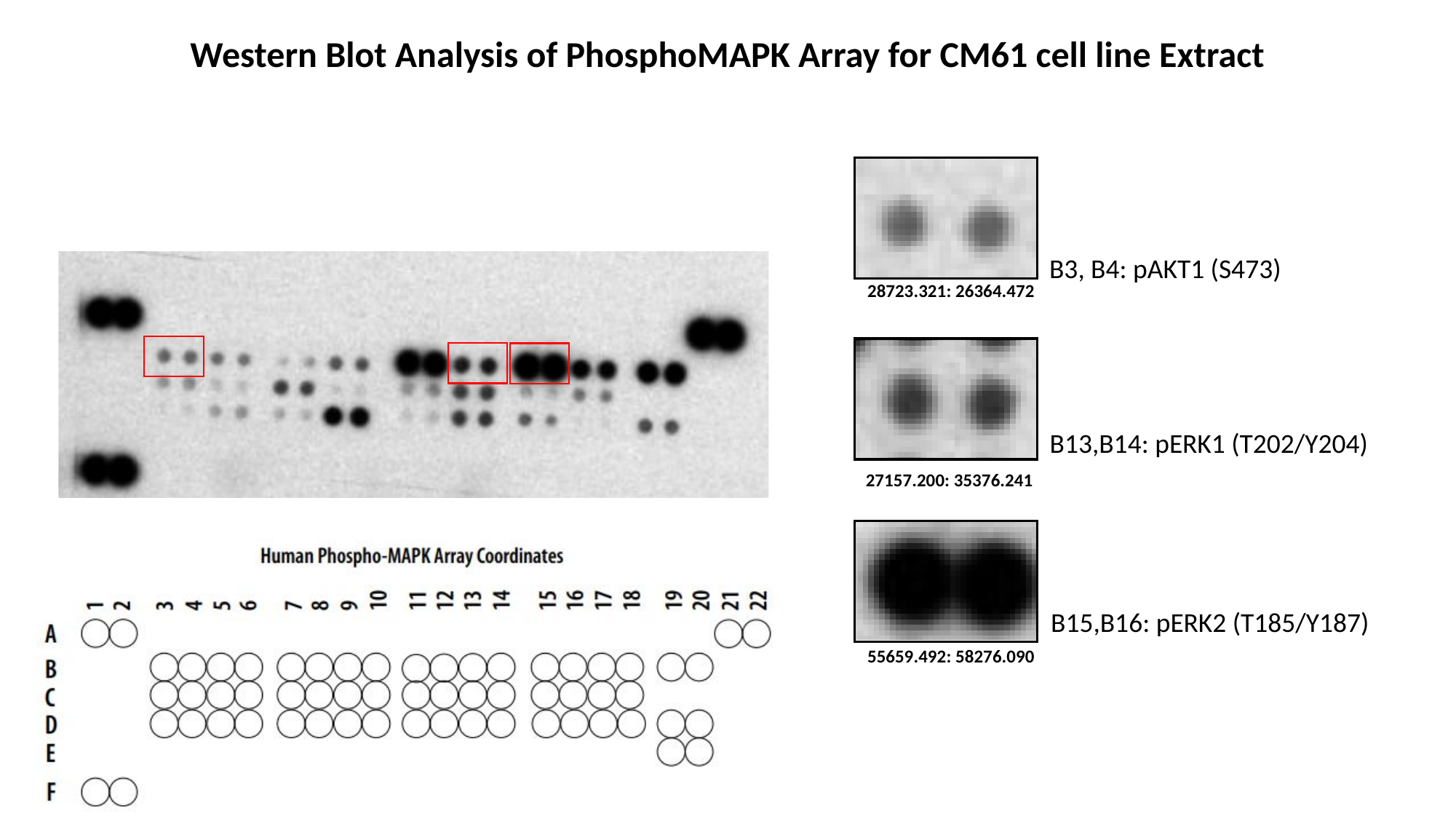

Western Blot Analysis of PhosphoMAPK Array for CM61 cell line Extract
B3, B4: pAKT1 (S473)
28723.321: 26364.472
B13,B14: pERK1 (T202/Y204)
27157.200: 35376.241
B15,B16: pERK2 (T185/Y187)
55659.492: 58276.090

## Slide 6
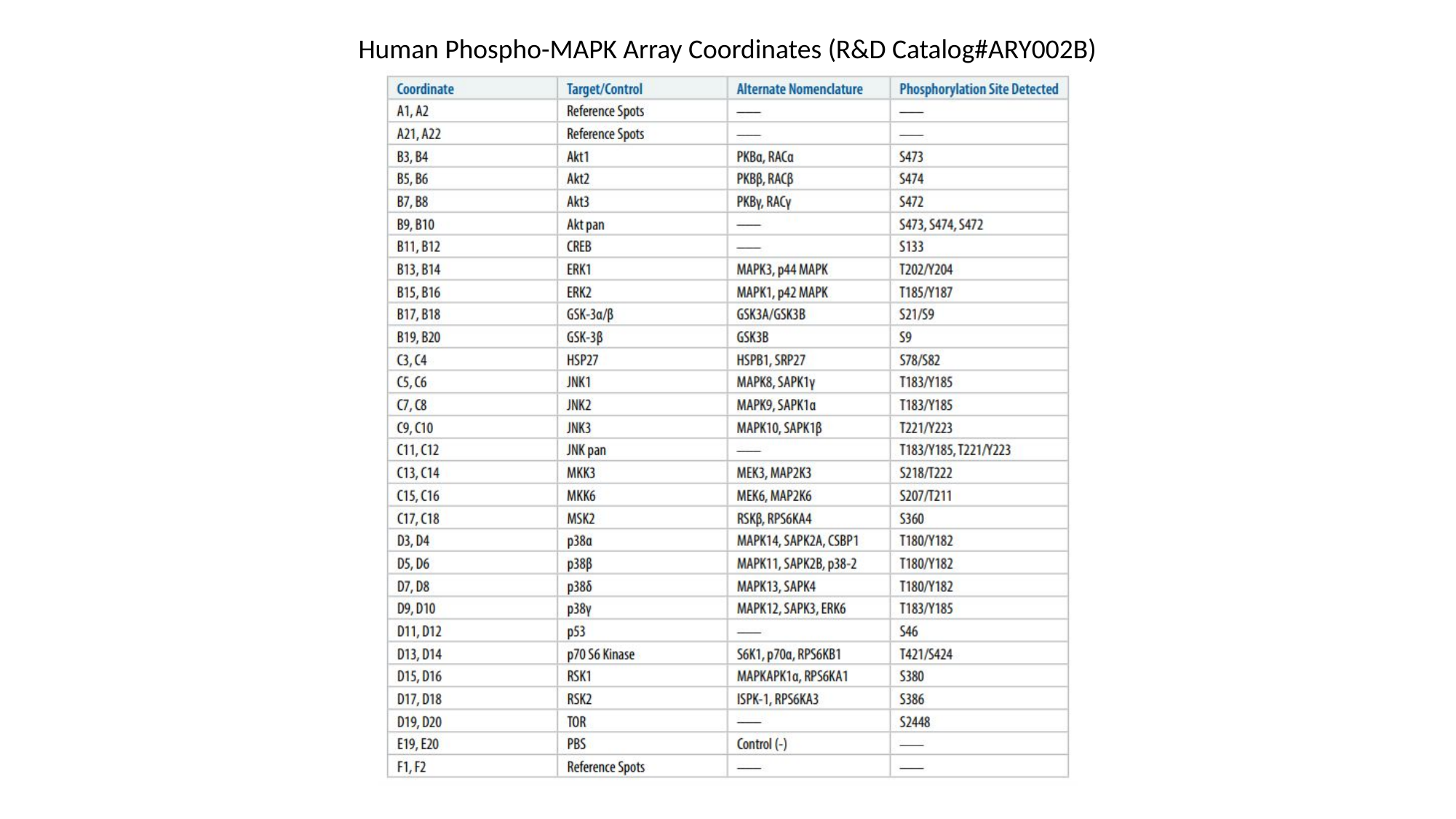

Human Phospho-MAPK Array Coordinates (R&D Catalog#ARY002B)

## Slide 7
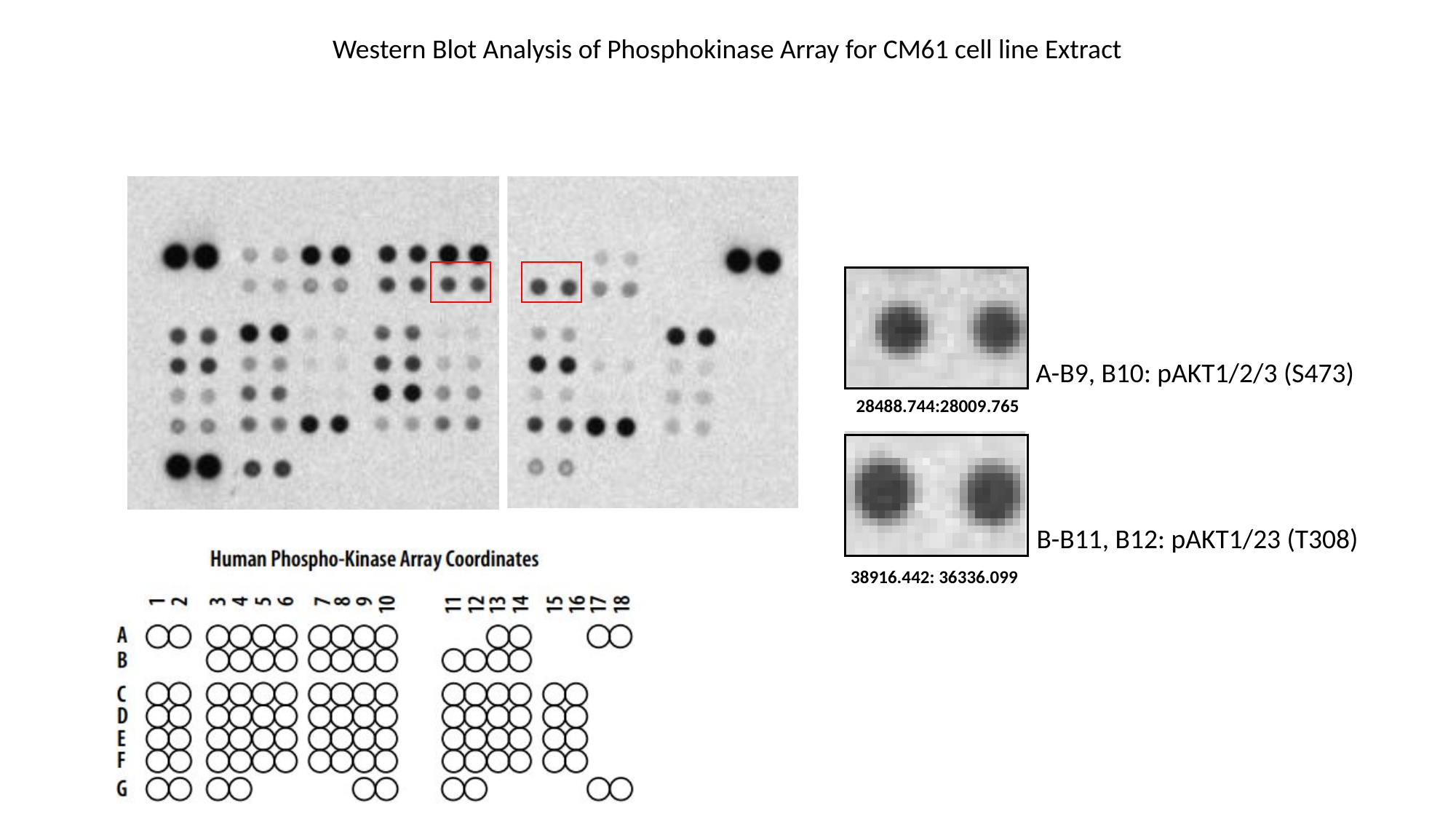

Western Blot Analysis of Phosphokinase Array for CM61 cell line Extract
A-B9, B10: pAKT1/2/3 (S473)
28488.744:28009.765
B-B11, B12: pAKT1/23 (T308)
38916.442: 36336.099

## Slide 8
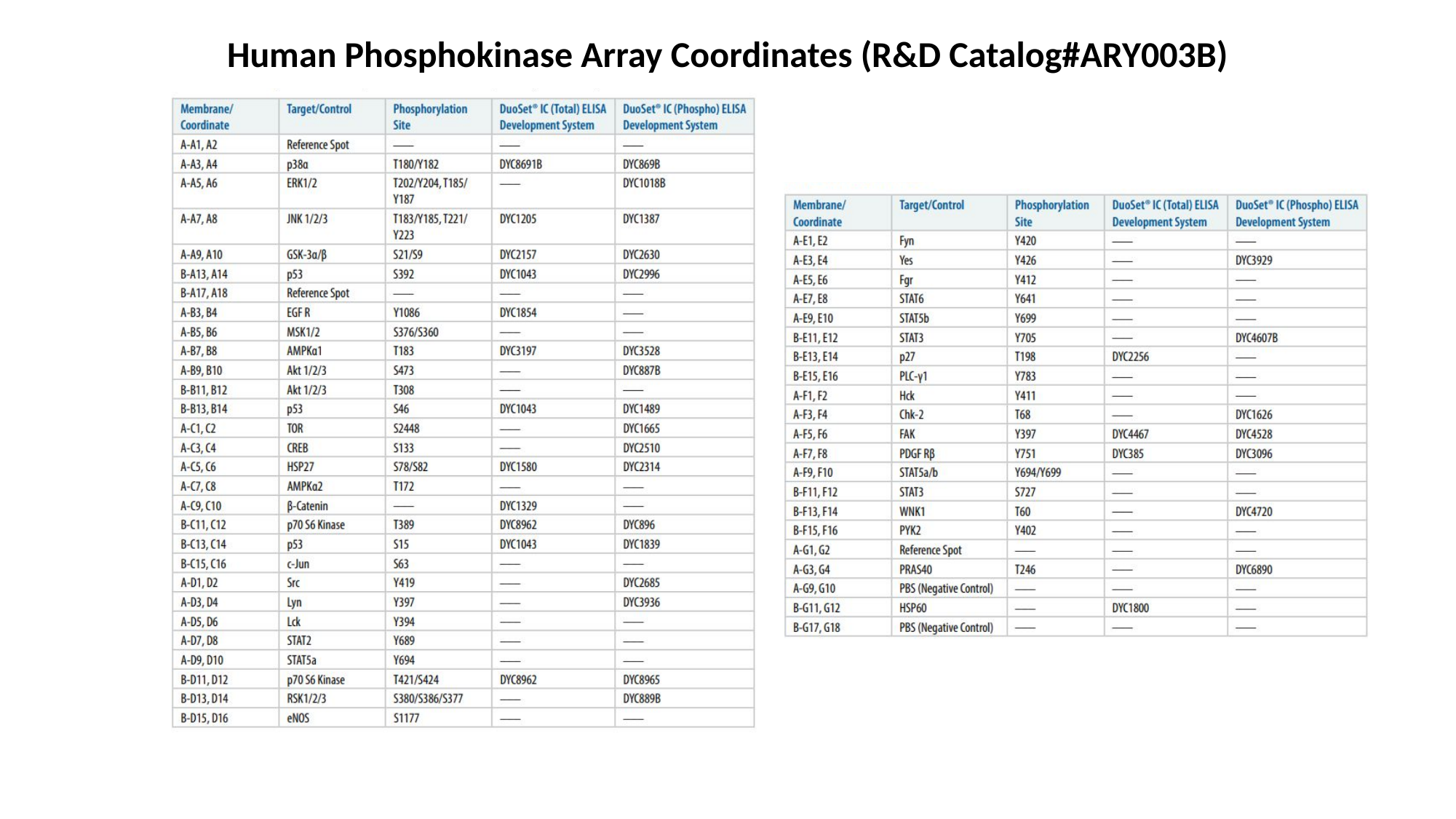

Human Phosphokinase Array Coordinates (R&D Catalog#ARY003B)

## Slide 9
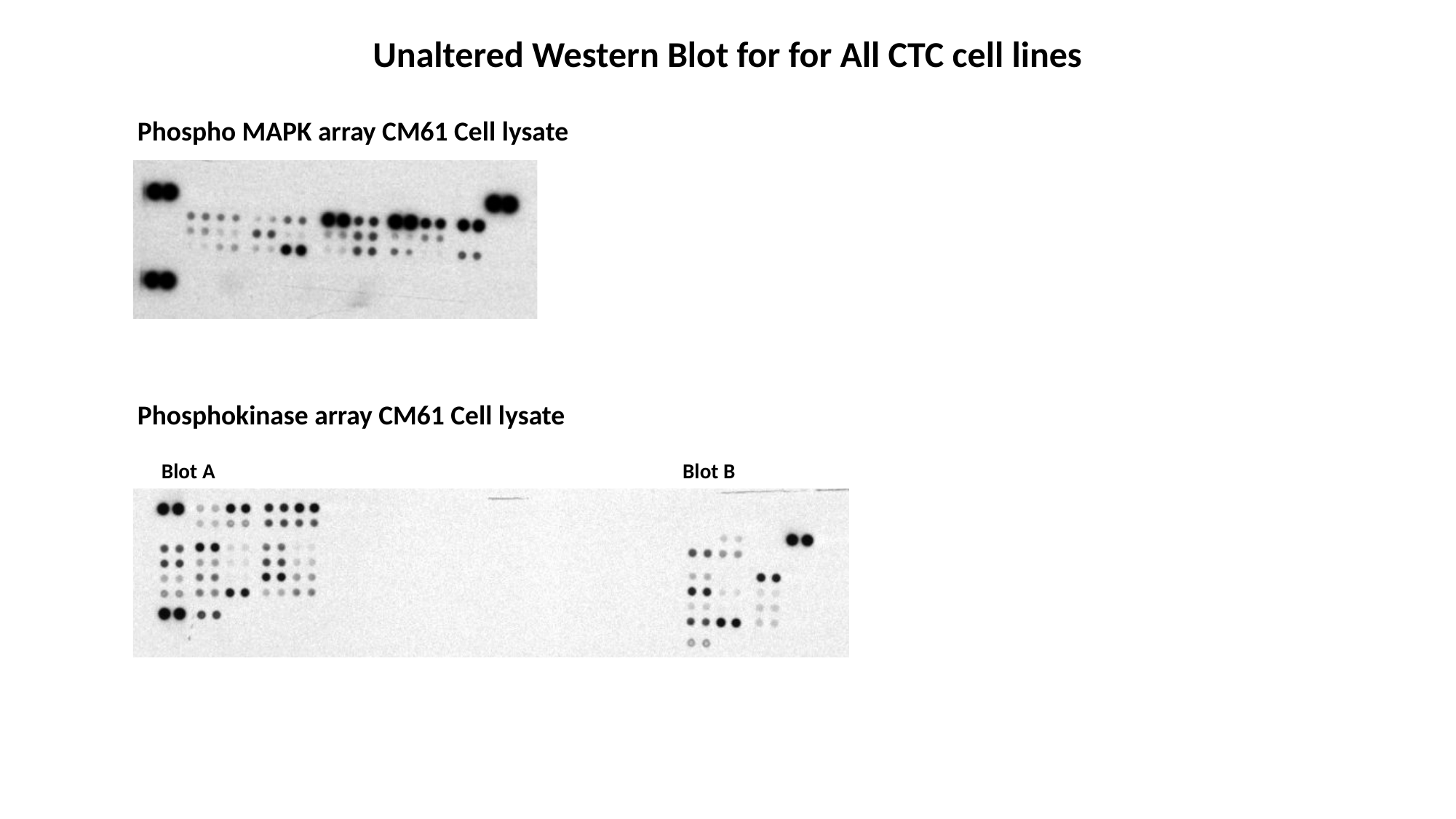

Unaltered Western Blot for for All CTC cell lines
Phospho MAPK array CM61 Cell lysate
Phosphokinase array CM61 Cell lysate
Blot A Blot B

## Slide 10
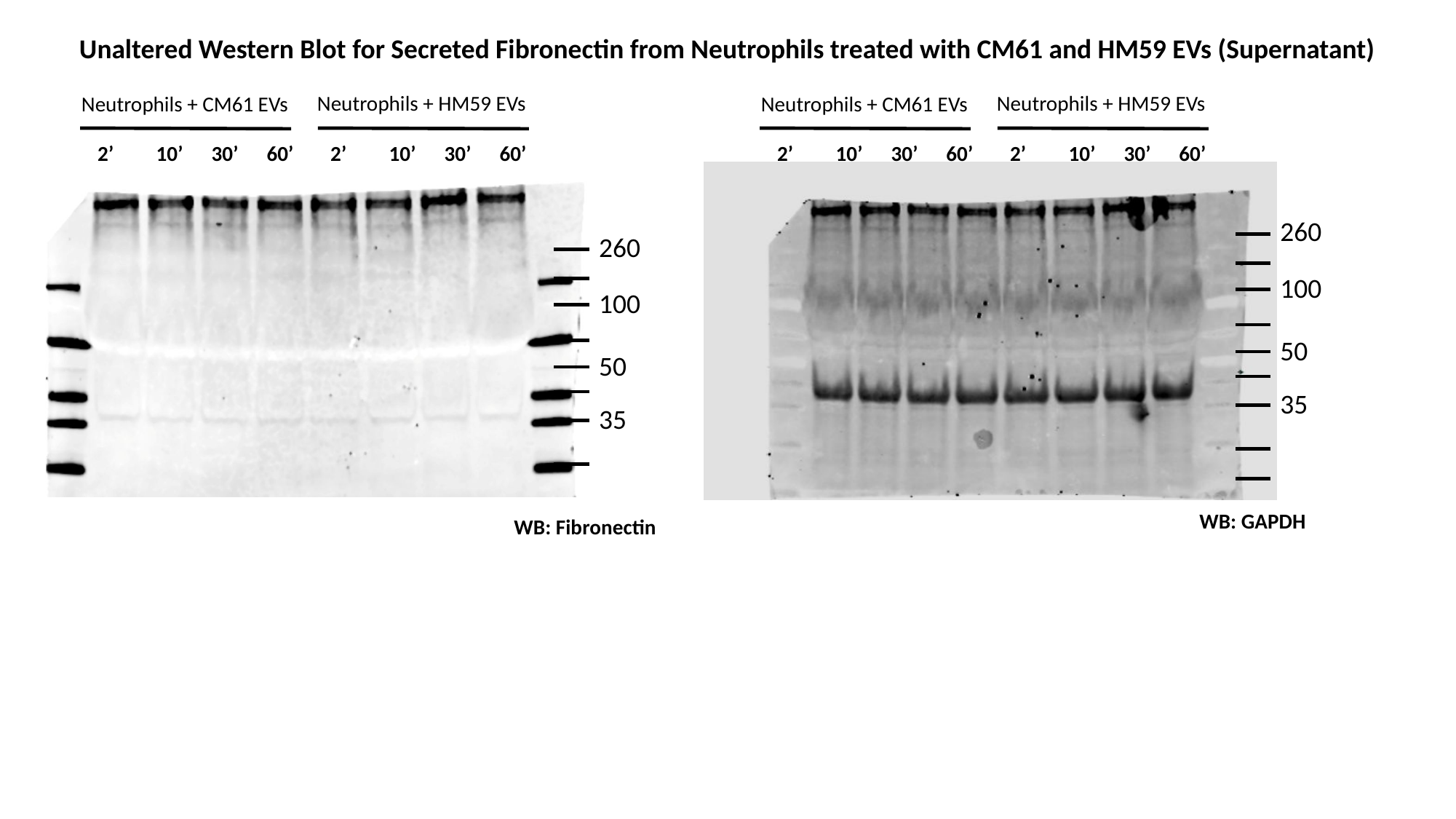

Unaltered Western Blot for Secreted Fibronectin from Neutrophils treated with CM61 and HM59 EVs (Supernatant)
Neutrophils + HM59 EVs
Neutrophils + CM61 EVs
2’ 10’ 30’ 60’
2’ 10’ 30’ 60’
Neutrophils + HM59 EVs
Neutrophils + CM61 EVs
2’ 10’ 30’ 60’
2’ 10’ 30’ 60’
260
100
50
35
260
100
50
35
WB: Fibronectin
WB: GAPDH
WB: Fibronectin

## Slide 11
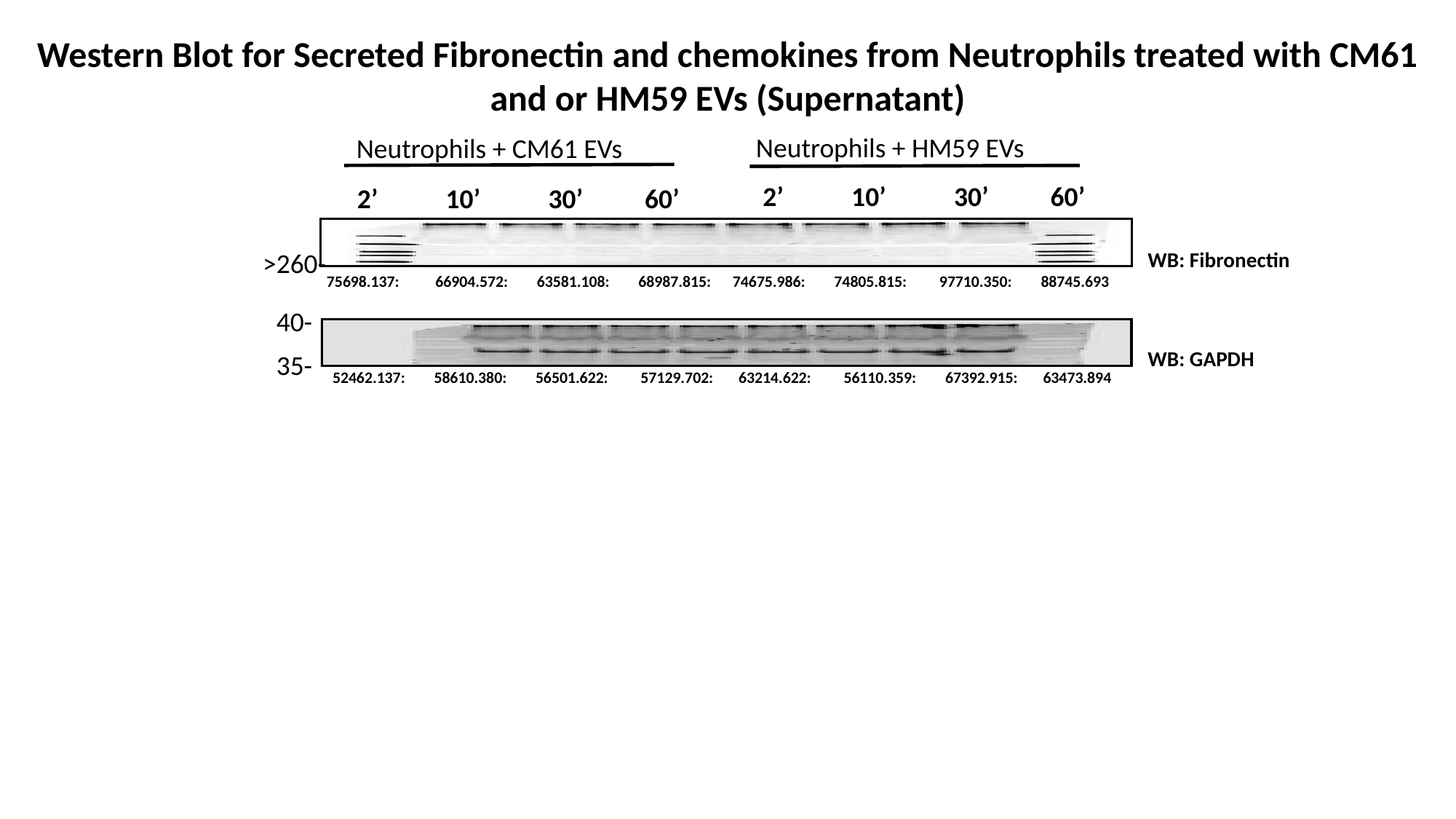

Western Blot for Secreted Fibronectin and chemokines from Neutrophils treated with CM61 and or HM59 EVs (Supernatant)
Neutrophils + HM59 EVs
Neutrophils + CM61 EVs
2’ 10’ 30’ 60’
2’ 10’ 30’ 60’
WB: Fibronectin
>260-
75698.137: 66904.572: 63581.108: 68987.815: 74675.986: 74805.815: 97710.350: 88745.693
40-
35-
WB: GAPDH
52462.137: 58610.380: 56501.622: 57129.702: 63214.622: 56110.359: 67392.915: 63473.894

## Slide 12
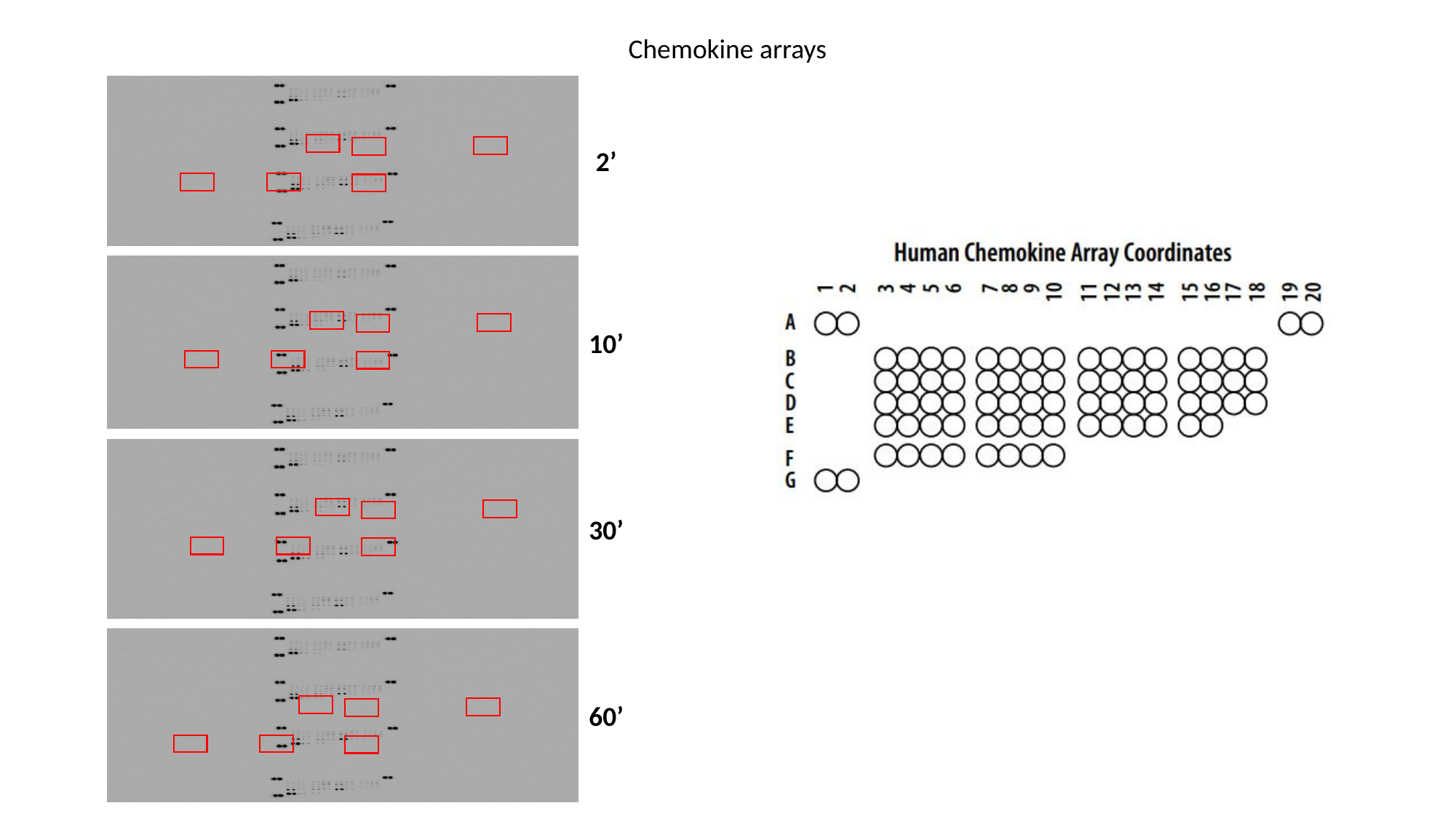

Chemokine arrays
2’
10’
30’
60’

## Slide 13
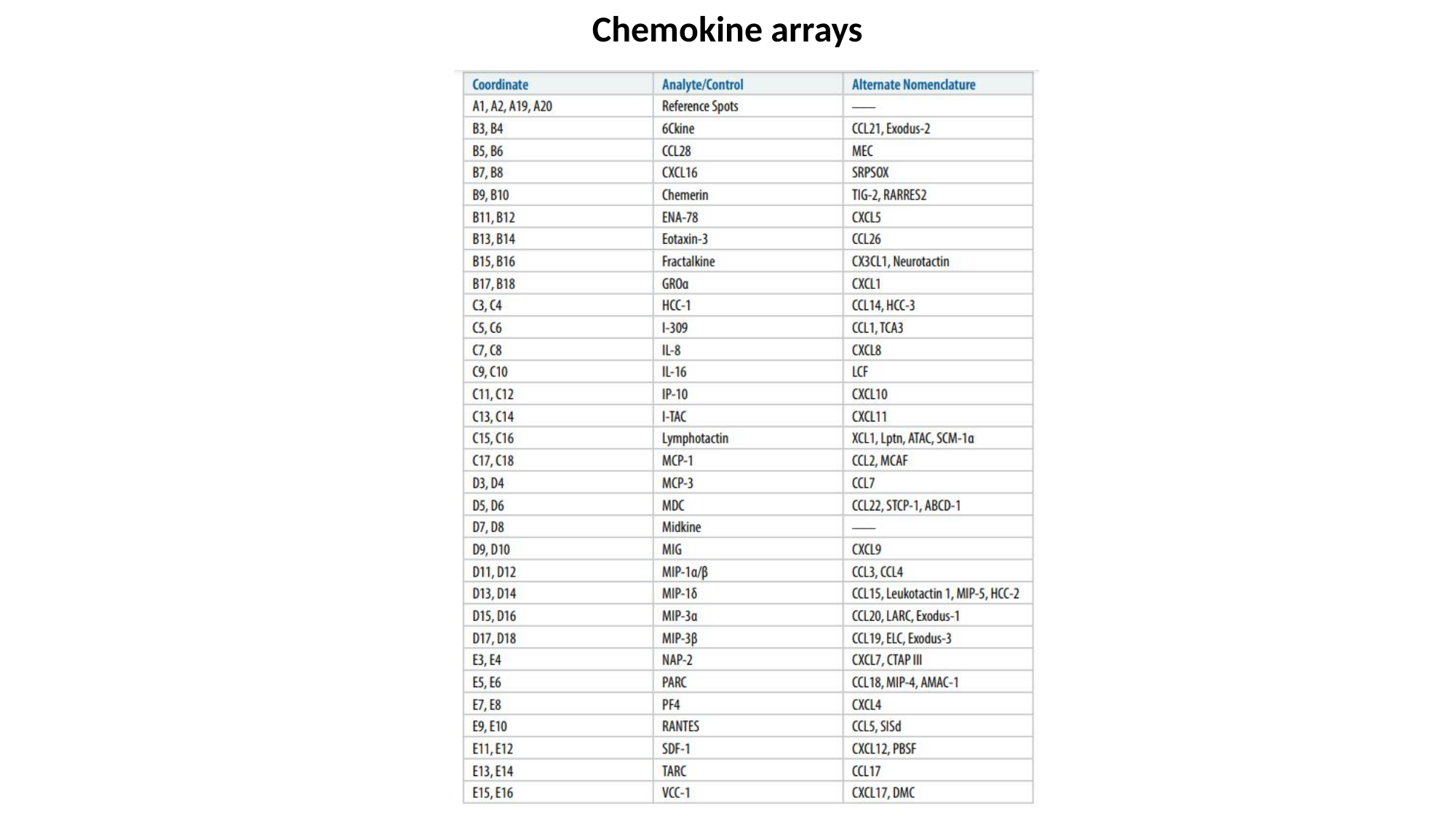

Chemokine arrays

## Slide 14
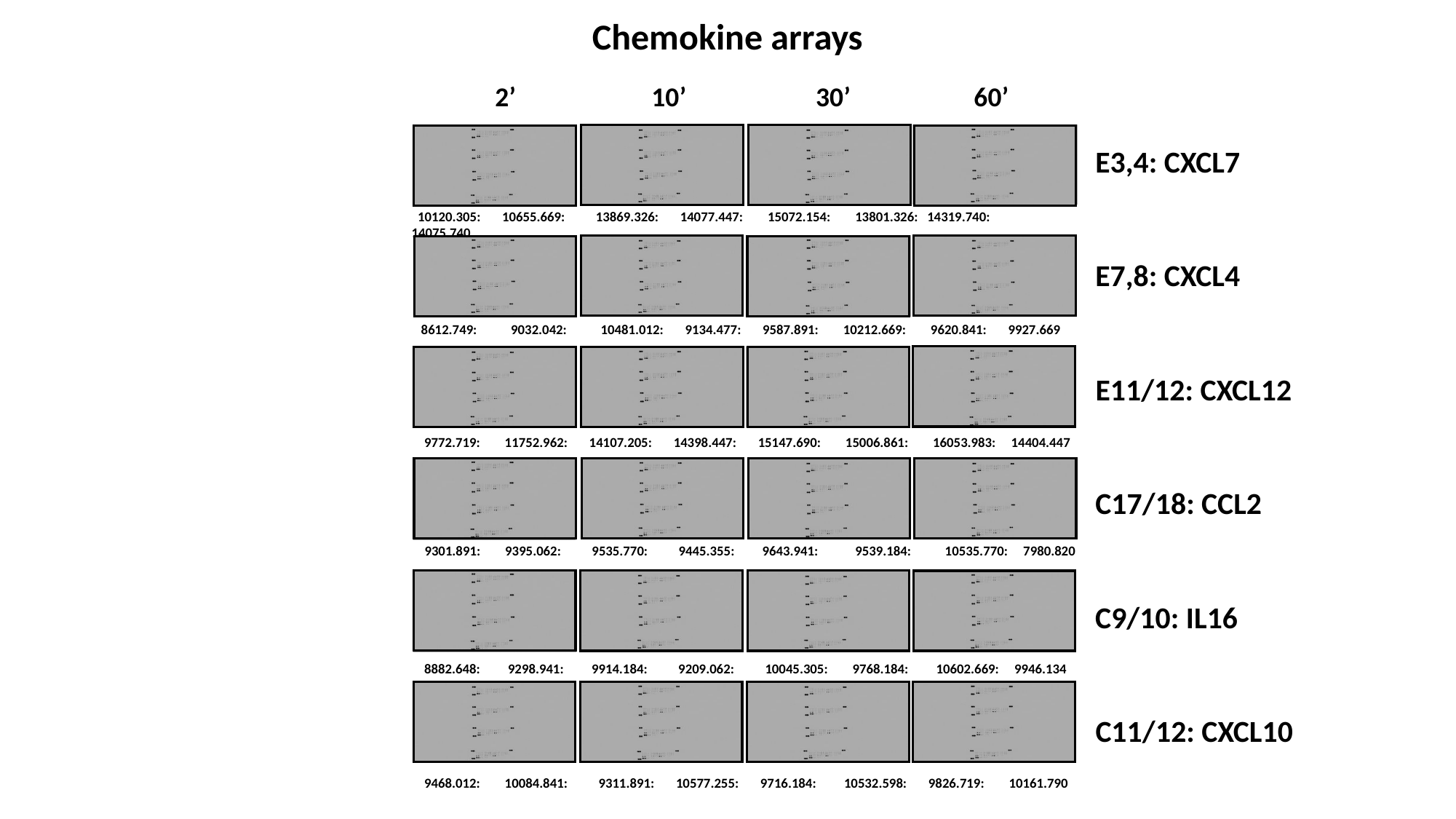

Chemokine arrays
2’ 10’ 30’ 60’
E3,4: CXCL7
 10120.305: 10655.669: 13869.326: 14077.447: 15072.154: 13801.326: 14319.740: 14075.740
E7,8: CXCL4
8612.749: 9032.042: 10481.012: 9134.477: 9587.891: 10212.669: 9620.841: 9927.669
E11/12: CXCL12
9772.719: 11752.962: 14107.205: 14398.447: 15147.690: 15006.861: 16053.983: 14404.447
C17/18: CCL2
9301.891: 9395.062: 9535.770: 9445.355: 9643.941: 9539.184: 10535.770: 7980.820
C9/10: IL16
8882.648: 9298.941: 9914.184: 9209.062: 10045.305: 9768.184: 10602.669: 9946.134
C11/12: CXCL10
9468.012: 10084.841: 9311.891: 10577.255: 9716.184: 10532.598: 9826.719: 10161.790

## Slide 15
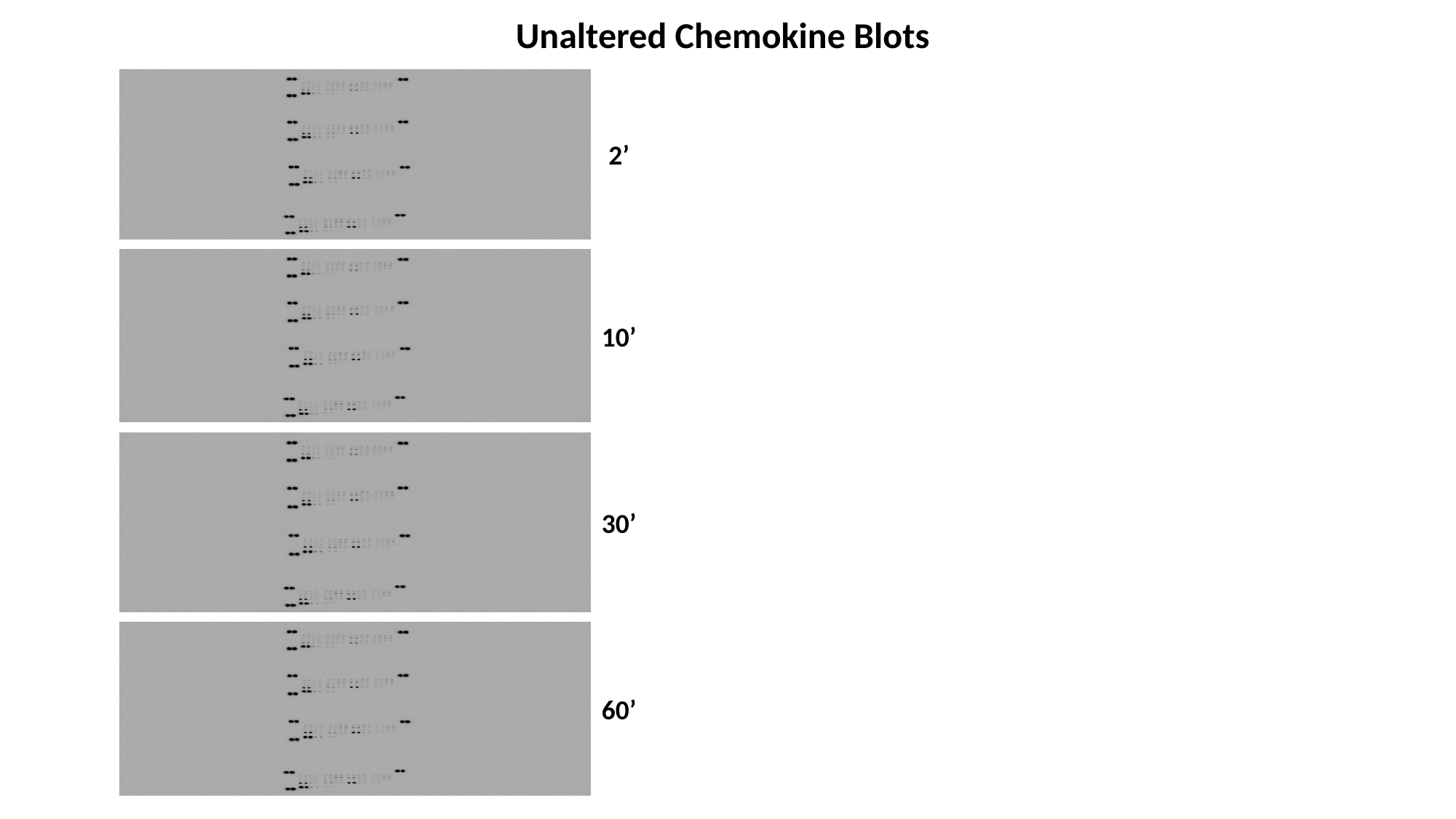

Unaltered Chemokine Blots
2’
10’
30’
60’
